# Value certainty and choice confidence are multidimensional constructs that guide decision-making

**DOI:** 10.3758/s13415-022-01054-4

**Published:** 2023-01-11

**Authors:** Douglas G. Lee, Todd A. Hare

**Affiliations:** 1 School of Psychological Sciences, Tel Aviv University, Tel Aviv-Yafo, Israel; 2 Zurich Center for Neuroeconomics, Department of Economics, University of Zurich, Zürich, Switzerland; 3 Neuroscience Center Zurich, University of Zurich and ETH Zurich, Zürich, Switzerland

**Keywords:** Multiattribute choice, Metacognition, Subjective value

## Abstract

The degree of certainty that decision-makers have about their evaluations of available choice alternatives and their confidence about selecting the subjectively best alternative are important factors that affect current and future value-based choices. Assessments of the alternatives in a given choice set are rarely unidimensional; their values are usually derived from a combination of multiple distinct attributes. For example, the taste, texture, quantity, and nutritional content of a snack food may all be considered when determining whether to consume it. We examined how certainty about the levels of individual attributes of an option relates to certainty about the overall value of that option as a whole and/or to confidence in having chosen the subjectively best available option. We found that certainty and confidence are derived from unequally weighted combinations of attribute certainties rather than simple, equal combinations of all sources of uncertainty. Attributes that matter more in determining choice outcomes also are weighted more in metacognitive evaluations of certainty or confidence. Moreover, we found that the process of deciding between two alternatives leads to refinements in both attribute estimations and the degree of certainty in those estimates. Attributes that are more important in determining choice outcomes are refined more during the decision process in terms of both estimates and certainty. Although certainty and confidence are typically treated as unidimensional, our results indicate that they, like value estimates, are subjective, multidimensional constructs.

**Supplementary Information:**

The online version contains supplementary material available at 10.3758/s13415-022-01054-4.

## Introduction

Most human behavior is guided, at some level, by subjective assessments of value or goal relevance. Explicitly evaluating options and/or choosing between two or more options according to their desirability are perhaps the most illustrative examples of this process. Even for decisions where the objective values are clearly defined and not malleable, the personal sense of the value of the options to individual decision-makers can nevertheless be subjective. Decisions where no universal objective values exist—perhaps the most common variety—must rely solely on subjective values. Previous research has established that people often subjectively assess choice options in terms of specific individual value-related attributes (Amasino et al., 2019; Barakchian et al., 2021; Bhatia and Stewart, 2018; Hare et al., 2011; Harris et al., 2018; Lee and Hare, 2022; Luce et al., 2000; Maier et al., 2020; Noguchi and Stewart, 2018; Reeck et al., 2017; Sullivan et al., 2015; Weber and Johnson, 2009). It is assumed that such attribute assessments should aggregate to approximate subjective estimates of overall value, provided that a sufficient set of attributes is considered. However, recent work has shown that the subjective assessments of individual attributes that people report influence decision behavior beyond the influence of aggregated overall value estimates. Specifically, computational models that incorporate measures of individual attributes in addition to or instead of measures of overall value provide better accounts for choice outcomes, response times, response movement trajectories, and confidence reports (Amasino et al., 2019; Lee, D’Alessandro, et al., 2022a; Lee and Hare, 2022; Yang and Krajbich, 2022).

Confidence reports often are used to examine how people evaluate their own performance on cognitive tasks, including decision-making, based solely on their own internal cognitive processes (i.e., not on external feedback). This ability is generally referred to as *metacognition*. Metacognitive assessments of choices are thought to aid in both current (Desender et al., 2021; Lee, Daunizeau, et al., 2022b; Lee and Daunizeau, 2021; van den Berg et al., 2016) and future decisions (Fleming and Daw, 2017; Yeung and Summerfield, 2012). For decisions with objectively correct responses, the degree to which confidence correlates with accuracy provides measures of metacognitive sensitivity and efficiency (Fleming and Lau, 2014). Metacognitive skills have been linked to individual differences in psychiatric symptomatology, radical political beliefs, intelligence, and academic performance (Atiya et al., 2021; Hoven et al., 2019; Ohtani and Hisasaka, 2018; Rollwage et al., 2018; Rouault et al., 2018). Thus, confidence and metacognition appear to have a substantial impact on a wide range of human thoughts and behaviors.

People can report choice confidence for decisions that are subjective (Brus et al., 2021; da Silva Castanheira et al., 2021; De Martino et al., 2013; Folke et al., 2016; Lee and Daunizeau, 2021; Quandt et al., 2022), as well as their level of certainty about self-reported estimates of subjective value (De Martino et al., 2013; Gwinn and Krajbich, 2020; Lee and Coricelli, 2020; Lee and Daunizeau, 2020, 2021; Polanía et al., 2019). The subjective measures of value certainty that people report have been shown to correlate systematically with key decision behavior, such as choice outcomes, response times, and choice confidence levels (Lee and Coricelli, 2020; Lee and Daunizeau, 2020, 2021). Specifically, when people report higher certainty about the sense of value that they have for the choice options (regardless of their actual value estimates), they choose more consistently, more quickly, and more confidently. This could be due to value signals in the brain either being enhanced by certainty or degraded by uncertainty as the choice options are compared (Brus et al., 2021; Lee and Usher, 2021). Nearly all studies of certainty and confidence in relation to value-based decisions have treated these metacognitive constructs as unidimensional.

The importance and impact of metacognitive evaluations of the individual attributes of choice options on valuation and decision-making is relatively unknown. We posit that value certainty and choice confidence form at the level of the individual attributes. We tested whether metacognitive evaluations of certainty about the overall value of an option or confidence in a decision outcome are based on equal aggregations of all sources of (un)certainty or instead come from unequally weighted combinations of certainty in option attributes. We also tested whether the certainty about option attributes increase over the course of deciding, and if so, whether that increase in certainty is equal for all attributes or if certainty about an attribute increases in proportion to the importance of that attribute for the decision outcome. We collected and analyzed data from two independent experiments. To briefly preview our results, we found that the contribution of an attribute to decision confidence as well as the amount of certainty that an attribute gains through the comparison process are both proportional to its importance in determining the decision outcome. Thus, like first-order constructions of value or goal relevance, second-order metacognitive evaluations of certainty or confidence are subjective combinations across attributes.

## Experiment 1

### Methods

#### Participants

A total of 107 people participated in the experiment (62 females; age: mean = 29 years, SD = 7, range 18-40). This sample size was chosen to be comparable to that used in previous studies based on a similar paradigm, while also allowing for sufficient sample sizes within each of the counterbalanced groups. All participants were recruited using Prolific (https://prolific.co). All were citizens of the United States or Canada, 101 were current residents of the United States or Canada, and 91 were native English speakers. Each participant received a payment of $10 as compensation for approximately 1 hour of time. Our experiments involved deidentified participant data, and protocols were approved by the Human Subjects Committee of the Faculty of Economics, Business Administration and Information Technology at the University of Zurich. All participants gave informed consent before commencing the experiments.

#### Materials

The experiments were constructed using the online experimental platform Gorilla (gorilla.sc). The experimental stimuli were drawn from a set of 200 digital images used in a previous study (Lee and Coricelli, 2020), each representing a distinct snack food item. Only the foods themselves were displayed in the images, no packaging or other materials were displayed. For this experiment, we used a subset of 100 stimuli, identical for all participants. We used the identical set of stimuli as was used for Experiment 1 in (Lee and Holyoak, 2021b), which had been determined by selecting the 100 items for which ratings from the original study varied the least (within each item) across participants.

#### Design and procedure

**Fig. 1:**
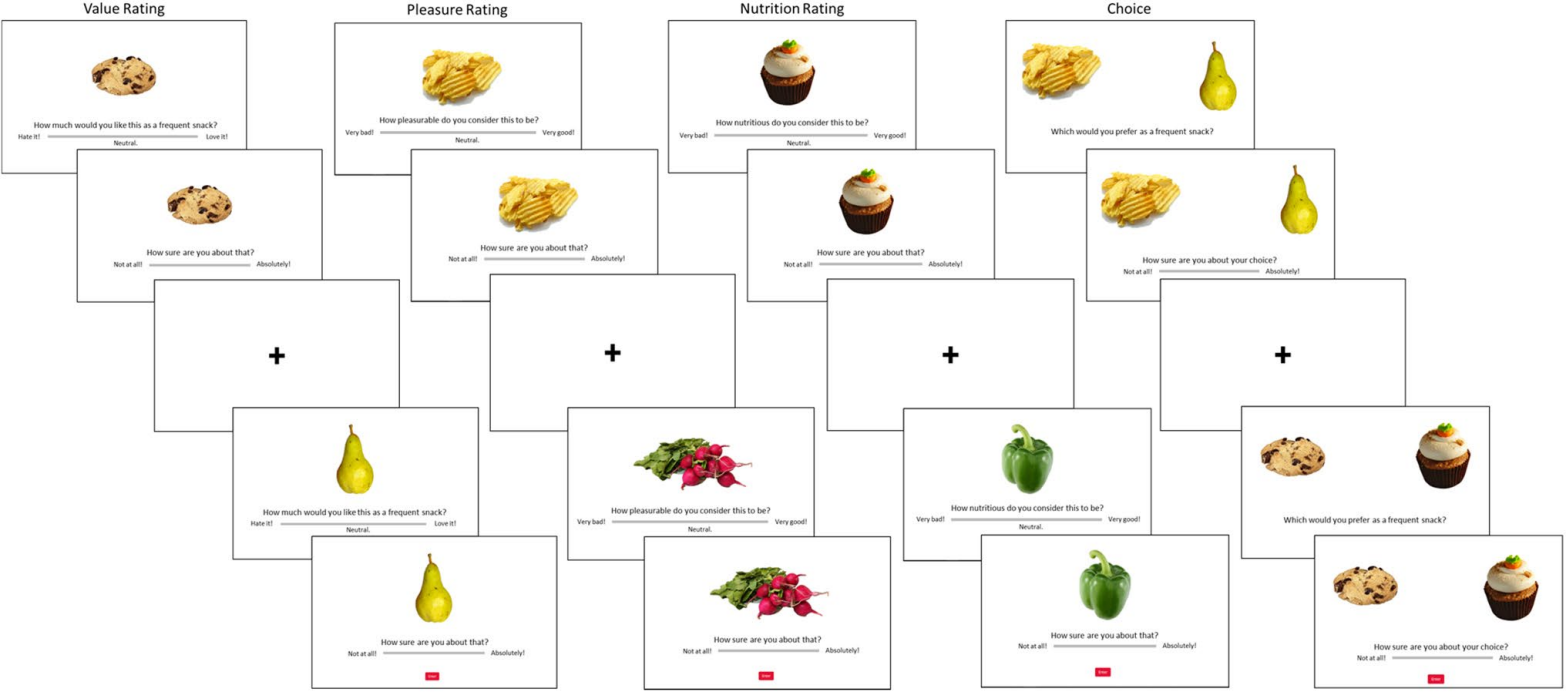
Task illustration. Example of what participants saw on the screen during each experimental section

The experiment consisted of a pre-exposure phase, followed by a rating phase, followed by a choice phase. No time limits were imposed for any of the constituent tasks, and a time limit of 140 minutes was imposed for the overall experiment (median completion time: 54 minutes). See Fig. 1 for an illustrative depiction of the flow of the experiment.

In the pre-exposure phase, participants simply observed as all individual items were displayed in a random sequence for 1,000 ms each. The purpose of the pre-exposure phase was to familiarize participants with the full set of items that they would later evaluate, allowing them to form an impression of the range of subjective values across the item set.

The rating phase comprised three sets of ratings—one for subjective overall value, and one for a subjective assessment of each of two individual attributes: pleasure and nutrition. In each rating task, all stimuli were displayed on the screen, one at a time, in a sequence randomized across participants. At the onset of each trial, the screen went blank for 100 ms; then, a fixation cross appeared at the center of the screen for 300 ms, and then the screen went blank again for 100 ms. Next, an image of a single food item appeared at the center of the screen. For the overall value rating, participants responded to the question, “How much would you want this as a frequent snack?” using a horizontal slider scale. This question was intended to motivate participants to think carefully while assessing the overall subjective quality of each option, as they should think more carefully when evaluating something they would consume frequently rather than just once.

Specifically, participants were instructed: “Click anywhere on the scale according to your personal preferences regarding eating the snack on a daily basis. The furthest left end of the scale indicates that you strongly dislike the snack and would hate to eat it often. The furthest right end of the scale indicates that you strongly like the snack and would love to eat it often. The middle of the scale indicates that you are neutral about eating the snack often. *Try to imagine committing to eating this item on a frequent basis, not just one single time.*” The leftmost end of the scale was labeled “Hate it!”; the center of the scale was labeled “Neutral”; and the rightmost end was labeled “Love it!” The scale appeared to participants to be continuous, and the data were captured in increments of 1 (range 1-100). There were no other marks on the scale, and the cursor did not appear on the scale until after participants clicked on it. Participants could revise their rating as many times as they liked before finalizing it. Participants clicked a button labeled “Enter” to finalize their overall value rating response and proceeded to the next screen. On the next screen, participants reported their subjective certainty about the overall value estimate that they had just reported. They responded to the question, “How sure are you about that?” using a continuous horizontal slider scale. The leftmost end of the scale was labeled “Not at all!” and the rightmost end was labeled “Absolutely!” The format of this slider scale was otherwise similar to the one for the overall value estimate—only half the overall length. Participants could revise their certainty as many times as they liked before finalizing it by clicking a button labeled “Enter.” The next trial then began.

In addition to overall value ratings, attribute ratings were performed separately for pleasure and nutrition, with the order counterbalanced across participants. The order of the overall value or attribute ratings also was counterbalanced across participants. One attribute was rated for every option (in one task section). Then, the other attribute was rated for every option (in the next task section). In each attribute rating task, all stimuli were displayed on the screen, one at a time, in a random sequence (randomized across participants and across sections for each participant). The format of the attribute rating tasks was identical to that of the overall value rating task, except now participants responded to the question, “How pleasurable do you consider this item to be?” or “How nutritious do you consider this item to be?” For the pleasure ratings, participants were instructed: “You will now evaluate the snack foods according to how pleasurable you believe them to be. Pleasure here refers to sensory enjoyment and can include whatever you think should be included, such as taste, smell, texture, appearance, etc.”

For the nutrition ratings, participants were instructed: “You will now evaluate the snack food items according to how nutritious you believe them to be. Nutrition can include whatever you think should be included, such as vitamins, minerals, protein, fat, carbohydrates, calories, etc.” For both attribute ratings, the leftmost end of the slider scale was labeled “Very bad!”; the center of the scale was labeled “Neutral”; and the rightmost end was labeled “Very good!” After finalizing their attribute rating for each option, participants then reported their subjective certainty about each attribute rating in a manner identical to that by which they reported the overall value certainty.

After completing all the rating task sections (overall value, pleasure, nutrition), participants completed the choice task. Before commencing the choice task, participants were instructed not to try to remember their earlier ratings, but rather to simply choose based on their current evaluations. For this task, 50 pairs of stimuli were displayed on the screen, one pair at a time, in a sequence randomized across participants. The pairings of items for each choice trial were identical to those used for Experiment 1 in Lee and Holyoak (2021b), which had been created to make the choices relatively difficult, as assessed by small differences in overall value ratings between the two items in each choice pair in the original study (Lee and Coricelli, 2020). Each individual item occurred in a single choice pair only. At the onset of each trial, the screen went blank for 100 ms, then a fixation cross appeared at the center of the screen for 300 ms, and then the screen went blank again for 100 ms. Next, a pair of images of food items appeared on the screen: one to the left and one to the right of the center of the screen. Participants responded to the question, “Which would you prefer as a frequent snack?” by clicking on the image of their preferred item. Participants then responded to the question, “How sure are you about your choice?” using a horizontal slider scale. The leftmost end of the scale was labeled “Not at all!” and the rightmost end was labeled “Absolutely!” Participants could revise their confidence report as many times as they liked before finalizing it. Participants clicked a button labeled “Enter” to finalize their confidence report and proceed to the next screen.

#### Statistical analyses

Unless stated otherwise, all statistical effect sizes reported below were calculated based on mixed effects (MFX) general linear model (GLM) regression analysis. The regressors of interest (as reported for each analysis below) were included as fixed-effects variables. All regressions also included random-effects slope and intercept terms for individual participants. All reported *p*-values are based on two-tailed tests. All rating variables were rescaled to the (−1,1] interval and all certainty and confidence variables were rescaled to the (0,1] interval before entering the analyses. To assist with both readability and interpretation, for all regression analyses except those related to choice consistency, we coded all variables such that option 1 (for each choice) refers to the option with the higher overall value rating. We thus defined overall value difference (dV) as the difference in overall value ratings (option 1 minus option 2). We defined pleasure difference (dP) and nutrition difference (dN) in an analogous manner. For the regressions related to choice consistency, we defined dV as the overall value of the option displayed on the right of the screen minus the overall value of the option displayed on the left of the screen (and similarly for dP, dN, dVC, dPC, and dNC) and defined consistency as the probability of choosing the option on the right. All response times (RTs) were log-transformed before entering the analyses. Within participant, we excluded trials with outlier RT (which we defined as log(RT) > median(log(RT)) + 3 * median_average_deviation(log(RT)) or log(RT) < median(log(RT)) - 3 * median_average_deviation(log(RT))).

### Results

#### Overall value estimates and certainty are composed of attribute estimates and certainty

**Fig. 2:**
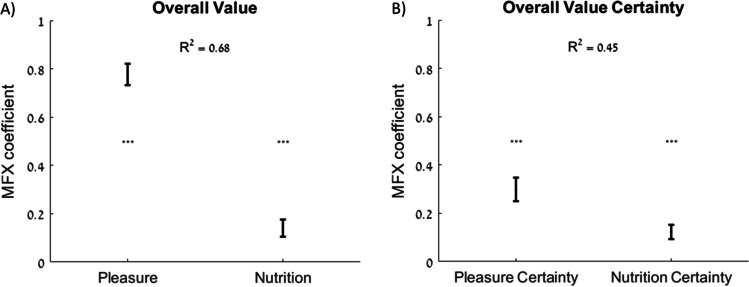
Contribution of attribute estimates and certainty to overall value estimates and certainty. **A**) Pleasure and nutrition ratings have a predictive effect on overall value ratings. **B**) Pleasure certainty and nutrition certainty have a predictive effect on overall value certainty. Error bars represent 95% confidence intervals. Significance stars: ***p < 0.001

How are attribute-level estimates and their certainty related to estimates and certainty for overall value? We addressed this question by first regressing overall value ratings on pleasure and nutrition ratings. The MFX coefficients for both attributes were significantly greater than zero (pleasure: 0.78, *p* < 0.001; nutrition: 0.14, *p* < 0.001; adjusted R^2^ = 0.68; Fig. 2A). This demonstrates that pleasure and nutrition together explain a large amount of overall value, but there are likely other attributes that also influence subjective assessments of overall value. Next, we regressed overall value certainty on pleasure certainty and nutrition certainty. Both MFX coefficients were significantly greater than zero (pleasure certainty: 0.30, *p* < 0.001; nutrition certainty: 0.12, *p* < 0.001; adjusted R^2^ = 0.45; Fig. 2B). Note that more of the variance in overall value and overall value certainty is explained by pleasure (R^2^ from regression of overall value on pleasure alone = 0.63) and pleasure certainty (R^2^ from regression of overall value certainty on pleasure certainty alone = 0.43), respectively, than by nutrition (R^2^ from regression of overall value on nutrition alone = 0.27) and nutrition certainty (R^2^ from regression of overall value certainty on nutrition certainty alone = 0.35). In other words, the rank order of the contributions of attribute certainty to overall value certainty matches the rank order of the attribute contributions to overall value, indicating that both overall value and overall value certainty are subjective constructs and that lower-level attributes contribute similarly to the estimation of each construct.

#### Choice behavior can be explained by attribute estimates and certainty

As a basic validation of the overall value rating and choice data, we tested how well choices could be explained by the difference in the overall value ratings of the options in each choice pair. We found that choices were highly aligned with overall value difference: across participants, 77% of all choices were made in accordance with the overall value ratings. We also regressed (logistic) choice on overall value difference and found the MFX coefficient to be significantly greater than zero (0.83, *p* < 0.001; adjusted R^2^ = 0.32). Next, we tested how well choices could be explained using differences in pleasure and nutrition, instead of overall value difference. The MFX coefficients for each attribute were significantly greater than zero (pleasure difference: 0.87, *p* < 0.001; nutrition difference: 0.14, *p* < 0.001; adjusted R^2^ = 0.31).

Previous studies have demonstrated associations between dependent variables related to choice—consistency (choosing the option with the higher overall value rating), response time (RT), and choice confidence—and independent variables related to overall value—sum and difference of value estimates and sum and difference of value certainty of the options within each choice pair. Specifically, each of these independent variables correlated positively with consistency and confidence, and negatively with RT (Lee and Coricelli, 2020; Lee and Daunizeau, 2020, 2021; Lee and Usher, 2021; Shevlin et al., 2022; Smith and Krajbich, 2019). Another previous study has shown that, in addition to the independent variables related to overall value, an independent variable related to individual attributes—*disparity*—shows the same qualitative relationships (Lee and Holyoak, 2021b). Attribute disparity accounts for the fact that choice options could be composed of very different attribute estimates, even if they were rated with similar overall values (see Supplementary Material for details). We replicated most of the associations between the aforementioned independent and dependent variables in our data (see Supplementary Material). The fact that attribute disparity has clear associations with the dependent choice variables already indicates that information about attributes contributes to the decision process beyond the information contained in general assessments of overall value.

**Fig. 3:**
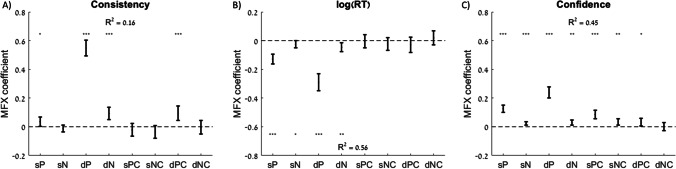
Explanatory power of attribute estimates and certainty on choice, response time, and confidence. Predictive effect of the sum of pleasure (sP), sum of nutrition (sN), difference in pleasure (dP), difference in nutrition (dN), sum of pleasure certainty (sPC), sum of nutrition certainty (sNC), difference in pleasure certainty (dPC), and difference in nutrition certainty (dNC) of the options on each trial on choice consistency (choosing the option with the higher overall value rating (**A**), response time (**B**), and choice confidence (**C**)). Error bars represent 95% confidence intervals. Significance stars: *p < 0.05; **p < 0.01; ***p < 0.001

Next, we examined how attribute-level measures might directly relate to choice consistency, RT, and confidence. We thus tested the associations between these three dependent variables and the sums of attribute estimates (both pleasure and nutrition), as well as the differences (which were previously examined in (Lee and Holyoak, 2021b)). We also went beyond previous work to determine whether, in addition to attribute estimates, attribute certainty was associated with choice consistency, RT, and confidence. Specifically, we tested the associations between these dependent variables and the sums of pleasure and nutrition certainty and the differences in pleasure and nutrition certainty (higher overall-valued option minus lower overall-valued option). We used three separate regression models to test the effects of all eight independent variables on the three dependent variables (Fig. 3; Table 1). In the regression with choice consistency as the dependent variable, we found significant positive associations with pleasure sum, pleasure difference, nutrition difference, and pleasure certainty difference (Fig. 3A). When RT was the dependent variable, there were significant negative associations with pleasure sum, nutrition sum, pleasure difference, and nutrition difference, but no significant associations with any of the certainty measures (Fig. 3B). When choice confidence was the dependent variable, there were significant positive associations with all attribute-level independent variables except nutrition certainty difference (Fig. 3C). Thus, we found that choice confidence was the most sensitive of the three dependent variables to measures of attribute certainty.

**Table 1:** Effects of rating estimates and certainty on choice consistency, response time, and confidence

	Consistency	log(RT)	Confidence
*pleasure sum (sP)*	0.04 (*p* = 0.041)	−0.13 (*p* < 0.001)	0.13 (*p* < 0.001)
*nutrition sum (sN)*	−0.01 (*p* = 0.307)	−0.02 (*p* = 0.048)	0.02 (*p* < 0.001)
*pleasure difference (dP)*	0.55 (*p* < 0.001)	−0.29 (*p* < 0.001)	0.24 (*p* < 0.001)
*nutrition difference (dN)*	0.09 (*p* < 0.001)	−0.05 (*p* = 0.003)	0.03 (*p* = 0.002)
*pleasure certainty sum (sPC)*	−0.02 (*p* = 0.335)	0.00 (*p* = 0.863)	0.09 (*p* < 0.001)
*nutrition certainty sum (sNC)*	−0.04 (*p* = 0.101)	−0.02 (*p* = 0.298)	0.03 (*p* = 0.003)
*pleasure certainty difference (dPC)*	0.09 (*p* < 0.001)	−0.03 (*p* = 0.286)	0.03 (*p* = 0.019)
*nutrition certainty difference (dNC)*	0.00 (*p* = 0.880)	0.02 (*p* = 0.421)	0.00 (*p* = 0.882)
	R^2^ = 0.16	R^2^ = 0.56	R^2^ = 0.45

Mixed-effects regression coefficients from three separate regressions using either choice consistency, response time, or confidence as the dependent variable. All regressions included random intercepts and slopes for each variable by participant

**Fig. 4:**
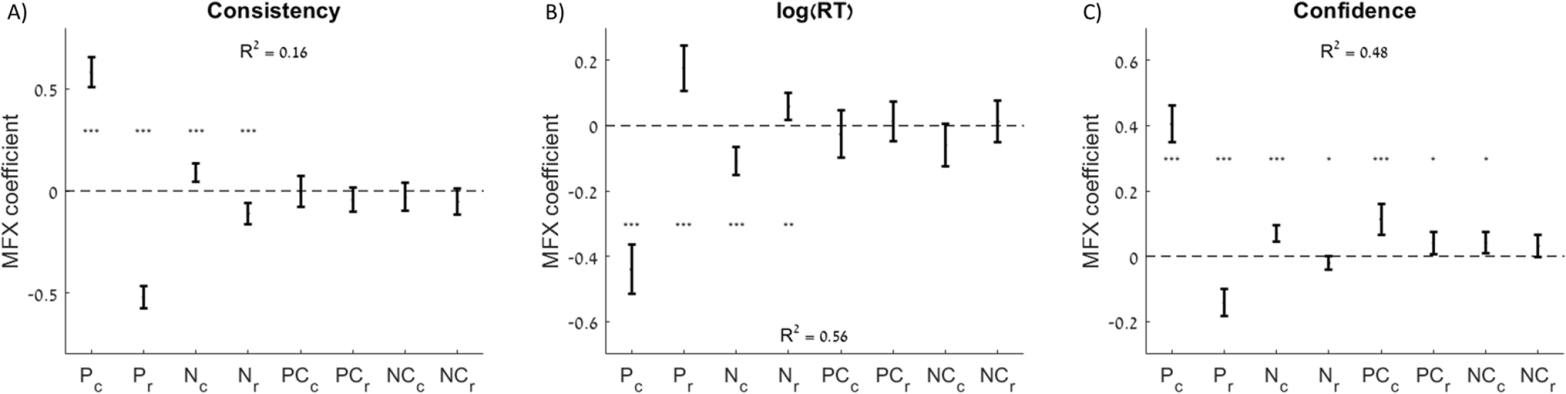
Explanatory power of attribute estimates and certainty on choice, response time, and confidence. Predictive effect of the pleasure, nutrition, pleasure certainty, and nutrition certainty for the chosen option (P_c_, N_c_, PC_c_, NC_c_) and for the rejected option (P_r_, N_r_, PC_r_, NC_r_) on each trial on choice consistency (choosing the option with the higher overall value rating (**A**), response time (**B**), and choice confidence (**C**)). Error bars represent 95% confidence intervals. Significance stars: *p < 0.05; **p < 0.01; ***p < 0.001.

We also tested the effects specific to the individual options on each trial (chosen or rejected), rather than sums or differences across options. Specifically, we tested the associations between the same three dependent variables (consistency, RT, and confidence) and the pleasure and nutrition estimate and certainty for the chosen and rejected options. We used three separate regression models to test the effects of all eight independent variables on the three dependent variables (Fig. 4; Table 2). In the regression with choice consistency as the dependent variable, we found significant positive associations with pleasure and nutrition for the chosen option, and significant negative associations with pleasure and nutrition for the rejected option, but no significant associations with any of the certainty terms (Fig. 4A). The magnitudes of the weights for chosen options were roughly equal to the weights for rejected options. When RT was the dependent variable, there were significant negative associations with pleasure and nutrition for the chosen option, and significant positive associations with pleasure and nutrition for the rejected option, but no significant associations with any of the certainty terms (although there appears to be a trend following the same pattern as for the other dependent variables; Fig. 4B). Interestingly, now the magnitudes of the weights for the chosen options were greater than the weights for the rejected options. When choice confidence was the dependent variable, there were significant positive associations with pleasure and nutrition for the chosen option and significant negative associations with pleasure and nutrition for the rejected option. There also were positive and mostly significant associations with all the certainty terms (Fig. 4C). It is interesting that choice confidence always increased with attribute certainty, regardless of whether it was for the chosen or rejected option. As with RT, the magnitudes of the weights for the chosen options were greater than for the rejected options.

**Table 2:** Effects of rating estimates and certainty on choice consistency, response time, and confidence

	MFX coefficients		
	Consistency	log(RT)	Confidence
*pleasure of chosen (P*_*c*_*)*	0.58 (*p* < 0.001)	−0.44 (*p* < 0.001)	0.40 (*p* < 0.001)
*pleasure of rejected (P*_*r*_*)*	−0.52 (*p* < 0.001)	0.18 (*p* < 0.001)	−0.14 (*p* < 0.001)
*nutrition of chosen (N*_*c*_*)*	0.09 (*p* < 0.001)	−0.11 (*p* < 0.001)	0.07 (*p* < 0.001)
*nutrition of rejected (N*_*r*_*)*	−0.11 (*p* < 0.001)	0.06 (*p* = 0.005)	−0.02 (*p* = 0.038)
*pleasure certainty of chosen (PC*_*c*_*)*	0 (*p* = 0.985)	−0.03 (*p* = 0.479)	0.11 (*p* < 0.001)
*pleasure certainty of rejected (PC*_*r*_*)*	−0.04 (*p* = 0.157)	0.01 (*p* = 0.679)	0.04 (*p* = 0.022)
*nutrition certainty of chosen (NC*_*c*_*)*	−0.03 (*p* = 0.411)	−0.06 (*p* = 0.076)	0.04 (*p* = 0.014)
*nutrition certainty of rejected (NC*_*r*_*)*	−0.05 (*p* = 0.117)	0.01 (*p* = 0.673)	0.03 (*p* = 0.076)
	R^2^ = 0.16	R^2^ = 0.56	R^2^ = 0.48

Table shows the mixed-effects regression coefficients from three separate regressions using either choice consistency, response time, or confidence as the dependent variable. All regressions included random intercepts and slopes for each variable by participant

### Discussion

In this first experiment, we examined participants’ subjective estimates of overall value, pleasure, and nutrition for a variety of different snack foods, as well as their subjective feelings of certainty about both their overall value estimates and their assessments of the individual attributes. Our results replicate the well-established finding that attributes are subjectively weighted when computing an overall value for an option. Beyond that, they also demonstrate that certainty about overall value is an unequally weighted combination of attribute-level certainty rather than an equal combination of certainty across the individual attributes. This indicates that metacognitive assessments, such as feelings of certainty about the subjective overall value estimates that one provides, originate with respect to individual attributes instead of, or in addition to, being derived from an aggregated overall value. Furthermore, in the context of our snack food choice paradigm, we showed that the rank order of the contributions of the pleasure and nutrition attribute estimates to overall value estimates matches the rank order of pleasure certainty and nutrition certainty to overall value certainty. This result further indicates that overall value judgments are a subjective agglomeration of attribute judgements, even at the metacognitive level.

Attribute judgements and their certainty also are subjectively related to binary choice consistency, response time, and confidence. We replicated previous findings that consistency, RT, and confidence relate to differences in the pleasure and nutrition attributes (Lee and Holyoak, 2021b) in the same way that many previous studies have shown that they relate to differences in overall value (Lee and Coricelli, 2020; Lee and Daunizeau, 2020, 2021; Lee and Usher, 2021; Shevlin et al., 2022; Smith and Krajbich, 2019). Furthermore, we showed that other aspects of individual attribute evaluations, such as the sum of estimates, correlated with all three dependent variables. In particular, choice confidence was enhanced by both the sums and differences of the attribute estimates, as well as by the sums and differences of the attribute certainty. Moreover, the regression weights for and variance explained (R^2^) by variables related to pleasure were greater than those for variables related to nutrition, which parallels the results showing the relative contributions of these attributes to overall value estimates and certainty. These results suggest that reported levels of confidence about choices are at least partially determined by unequally weighted subjective feelings of certainty about attribute estimates, in a manner similar to how choices themselves are driven to a greater extent by specific attributes.

## Experiment 2

### Methods

#### Participants

A total of 93 people participated in the experiment (44 female; age: mean = 28 years, SD = 6, range 18-40). This sample size was chosen to be comparable to that used in previous studies based on a similar paradigm, while allowing for sufficient sample sizes within each of the counterbalanced groups. All participants were recruited using Prolific (https://prolific.co). All were citizens of the United States or Canada, 88 were current residents of the United States or Canada, and 76 were native English speakers. Each participant received a payment of $10 as compensation for approximately one hour of time. Our experiments involved de-identified participant data, and protocols were approved by the Human Subjects Committee of the Faculty of Economics, Business Administration and Information Technology at the University of Zurich. All participants gave informed consent before commencing the experiments.

#### Materials

The experiments were constructed using the online experimental platform Gorilla (gorilla.sc). For this experiment, we used a subset of 60 stimuli (of the 100 used in Experiment 1), identical for all participants. We used the identical set of stimuli that was used for Experiments 2-5 in Lee and Holyoak (2021b), which had been determined by selecting from the larger subset of 100 (Experiment 1 of this study and Experiment 1 in Lee and Holyoak (2021b) the 60 items appearing in the 30 choice pairs that generated the highest overall spreading of alternatives (SoA) across participants in Experiment 1 of Lee and Holyoak (2021b).

#### Design and procedure

The experiment consisted of a pre-exposure phase, followed by an initial rating phase, followed by a choice phase, and followed by a final rating phase. No time limits were imposed for any of the constituent tasks. A time limit of 140 minutes was imposed for the overall experiment (median completion time: 51 minutes).

The pre-exposure phase was identical to that in Experiment 1, except with fewer stimuli. The initial rating phase was identical to the rating phase in Experiment 1, except with fewer stimuli. The choice phase was identical to that in Experiment 1, except with fewer pairs of stimuli (identical to those used for Experiments 2-5 Lee and Holyoak (2021b).

After completing the choice task, participants completed the final rating task. The final rating task was identical in format to the initial rating task. The sequence of rating sections (overall value, pleasure, nutrition) for each participant was the same in the final rating task as it had been in the initial rating task. For each participant, the stimuli were presented in a different random order than in the initial rating task. Before commencing the final rating task, participants were instructed not to try to remember their earlier ratings but rather to simply rate the stimuli as they now evaluated them.

#### Statistical analyses

All statistical analyses were performed in an identical manner as in Experiment 1.

### Results

Before testing for additional effects beyond those that we observed in the data from the first experiment, we first verified that each of the results that we reported for Experiment 1 replicated for Experiment 2:

#### Overall value estimates and certainty are composed of attribute estimates and certainty

**Fig. 5:**
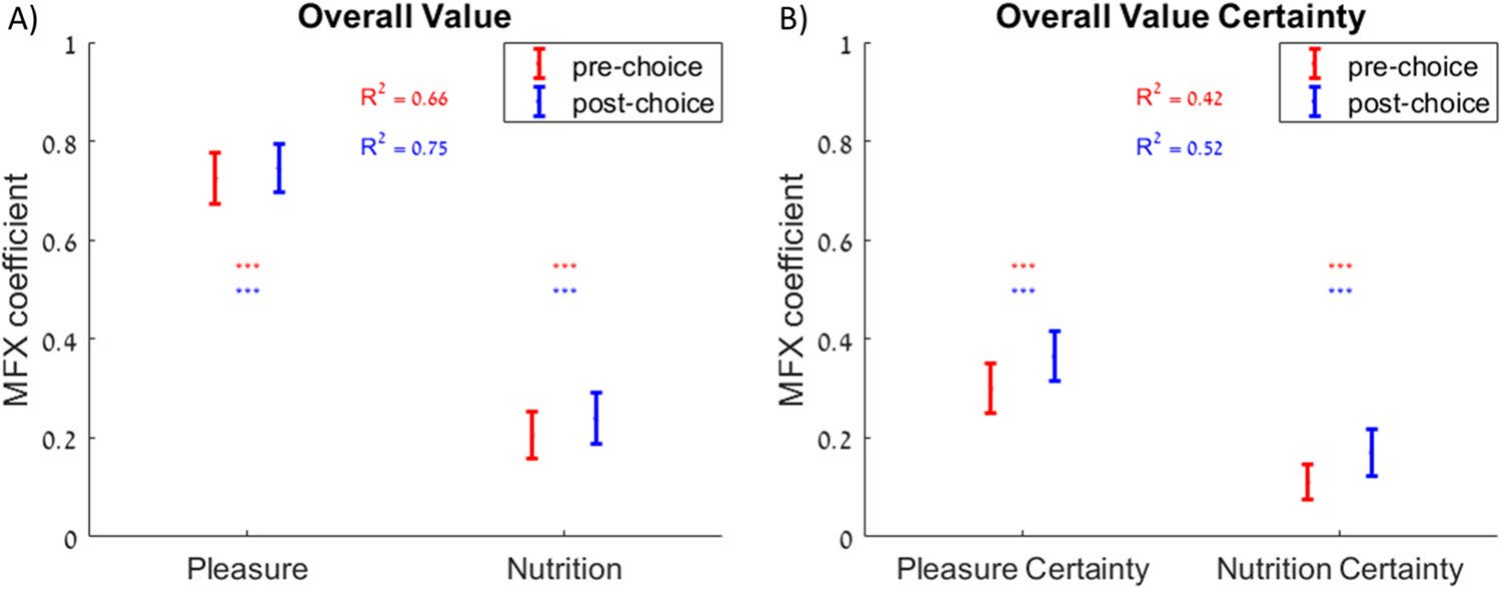
Contribution of attribute estimates and certainty to overall value estimates and certainty. **A**) Pleasure and nutrition ratings have a predictive effect on overall value ratings. **B**) Pleasure certainty and nutrition certainty have a predictive effect on overall value certainty. Error bars represent 95% confidence intervals. Significance stars: ***p < 0.001

We replicated the finding that both overall value estimates and certainty are subjectively weighted combinations of attribute estimates and certainty, respectively. Once again, we first regressed overall value ratings on pleasure and nutrition ratings. The MFX coefficients for both attributes were significantly greater than zero (pleasure: 0.72, *p* < 0.001; nutrition: 0.21, *p* < 0.001; adjusted R^2^ = 0.66; Fig. 5A in red). We then repeated the same regression analysis, this time using post-choice ratings instead of pre-choice. Post-choice ratings yielded MFX coefficients that were similar to (and slightly greater than) those for pre-choice ratings, and the post-choice ratings explained more of the variance (pleasure: 0.75, *p* < 0.001; nutrition: 0.24, *p* < 0.001; adjusted R^2^ = 0.75; Fig. 5A in blue). This supports the idea that participants refined their evaluations during choice deliberation and maintained those refined representations throughout the remainder of the experiment (Lee and Daunizeau, 2021; Lee and Holyoak, 2021a). To test whether the increase in R^2^ was statistically significant, we repeated the same regressions separately for each participant and examined the cross-participant R^2^ values for the regressions based on post-choice ratings versus those based on pre-choice ratings. The R^2^ values based on post-choice ratings were significantly greater, according to a Wilcoxon signed-rank test (mean difference in R^2^ = 0.11, *p* < 0.001).

We then regressed overall value certainty on pleasure certainty and nutrition certainty. Both MFX coefficients were significantly greater than zero (pleasure certainty: 0.30, *p* < 0.001; nutrition certainty: 0.11, *p* < 0.001; adjusted R^2^ = 0.42; Fig. 5B in red). Repeating the same regression analysis using post-choice ratings yielded MFX coefficients that were similar to (and slightly greater than) those for pre-choice ratings, and the post-choice ratings explained more of the variance (pleasure certainty: 0.36, *p* < 0.001; nutrition certainty: 0.17, *p* < 0.001; adjusted R^2^ = 0.52; Fig. 5B in blue). To test whether the increase in R^2^ was statistically significant, we repeated the same regressions separately for each participant and examined the cross-participant R^2^ values for the regressions based on post-choice ratings versus those based on pre-choice ratings. The R^2^ values based on post-choice ratings were significantly greater, according to a Wilcoxon signed rank test (mean difference in R^2^ = 0.05, *p* = 0.005).

Consistent with Experiment 1, more of the variance in overall value and overall value certainty is explained by pleasure (adjusted R^2^ from regression of overall value on pleasure alone = 0.57 or 0.64, using pre- or post-choice ratings, respectively) and pleasure certainty (adjusted R^2^ from regression of overall value certainty on pleasure certainty alone = 0.41 or 0.47, using pre- or post-choice ratings, respectively), respectively, than by nutrition (adjusted R^2^ from regression of overall value on nutrition alone = 0.26 or 0.30, using pre- or post-choice ratings, respectively) and nutrition certainty (adjusted R^2^ from regression of overall value certainty on nutrition certainty alone = 0.32 or 0.42, using pre- or post-choice ratings, respectively).

#### Choice behavior can be explained by attribute estimates and certainty

Choices were highly aligned with overall value differences. Across participants, 78% of all choices were made in accordance with the pre-choice overall value ratings. We regressed (logistic) choice on overall value difference and found the MFX coefficient to be significantly greater than zero (0.82, *p* < 0.001; adjusted R^2^ = 0.39). We also regressed (logistic) choice on pleasure difference and nutrition difference in the place of overall value difference. The MFX coefficients for each attribute were significantly greater than zero (pleasure difference: 0.79, *p* < 0.001; nutrition difference: 0.19, *p* < 0.001; adjusted R^2^ = 0.36). We then repeated the same analysis, this time using post-choice ratings. Choices were slightly more aligned with post-choice overall value difference (80% of all choices, across participants). Post-choice ratings yielded MFX coefficients that were similar to those for pre-choice ratings, and they explained more of the variance (overall value difference: 0.85, *p* < 0.001; adjusted R^2^ = 0.43; pleasure difference: 0.84, *p* < 0.001; nutrition difference: 0.15, *p* < 0.001; adjusted R^2^ = 0.40). To test whether the increase in R^2^ was statistically significant, we repeated the same regressions separately for each participant and examined the cross-participant R^2^ values for the regressions based on post-choice ratings versus those based on pre-choice ratings. For the regressions based on overall value, the R^2^ values were higher when using post- versus pre-choice ratings (mean difference = 0.08, *p* = 0.002). For the regressions based on individual attributes, the R^2^ values were not significantly different, according to a Wilcoxon signed rank test (mean difference = 0.02, *p* = 0.246).

Lastly, we note that our data replicate many previous results regarding the relationships between overall value sum and difference, overall value certainty sum and difference, and attribute disparity, and the dependent choice variables: consistency, RT, and confidence (see Supplementary Material). We also note that for these regressions based on overall value ratings and certainty as well as attribute disparity, the cross-participant R^2^ values were significantly greater for regressions based on post- versus pre-choice ratings, according to Wilcoxon signed rank tests (consistency: mean difference in R^2^ = 0.09, *p* = 0.015; RT: mean difference in R^2^ = 0.07, *p* = 0.009; confidence: mean difference in R^2^ = 0.04, *p* = 0.014).

**Fig. 6:**
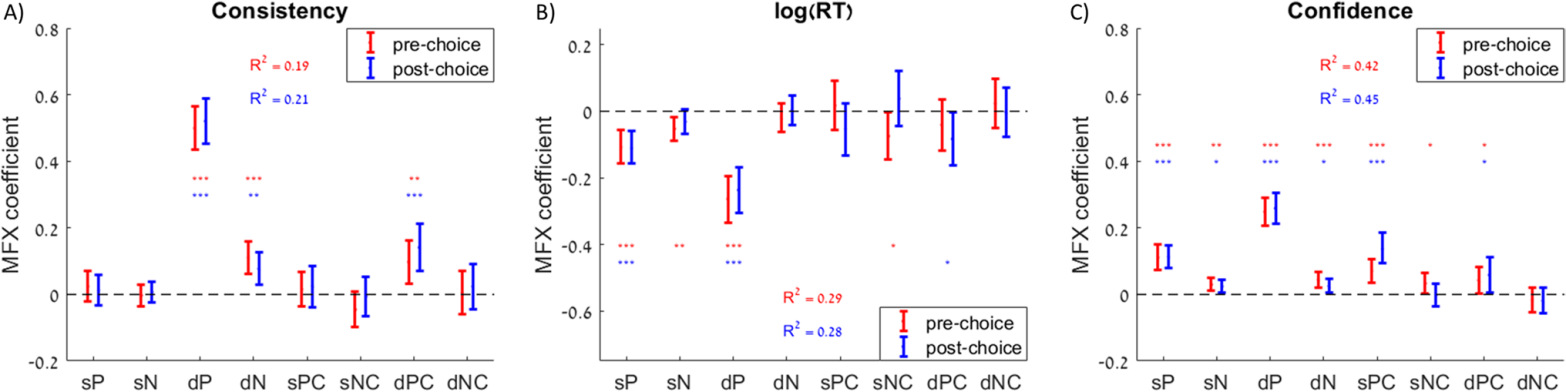
fig Explanatory power of attribute estimates and certainty on choice, response time, and confidence. Predictive effect of the sum of pleasure (sP), sum of nutrition (sN), difference in pleasure (dP), difference in nutrition (dN), sum of pleasure certainty (sPC), sum of nutrition certainty (sNC), difference in pleasure certainty (dPC), and difference in nutrition certainty (dNC) of the options on each trial on choice consistency (choosing the option with the higher overall value rating; A), response time (B), and choice confidence (C). Error bars represent 95% confidence intervals. Significance stars: *p < 0.05; **p < 0.01; ***p < 0.001

Choices also were strongly associated with attribute ratings and certainty. We regressed choice consistency, RT, and confidence (separately) on pleasure sum and difference, nutrition sum and difference, pleasure certainty sum and difference, and nutrition certainty sum and difference (Fig. 6 in red; Table 3). For choice consistency, we found positive associations with pleasure difference, nutrition difference, and pleasure certainty difference. For RT, we found negative associations with pleasure sum and difference, nutrition sum, and nutrition certainty sum. For confidence, we found positive associations with all variables except nutrition certainty difference. We then repeated the same regression analyses, this time using post-choice ratings. Post-choice ratings yielded MFX coefficients that were similar to those for pre-choice ratings (with a few changes in significance; Fig. 6 in blue; Table 3). Across participants, the R^2^ values based on pre- or post-choice ratings were not significantly different, according to Wilcoxon signed rank tests (*p* > 0.354 for all dependent variables).

**Table 3:**
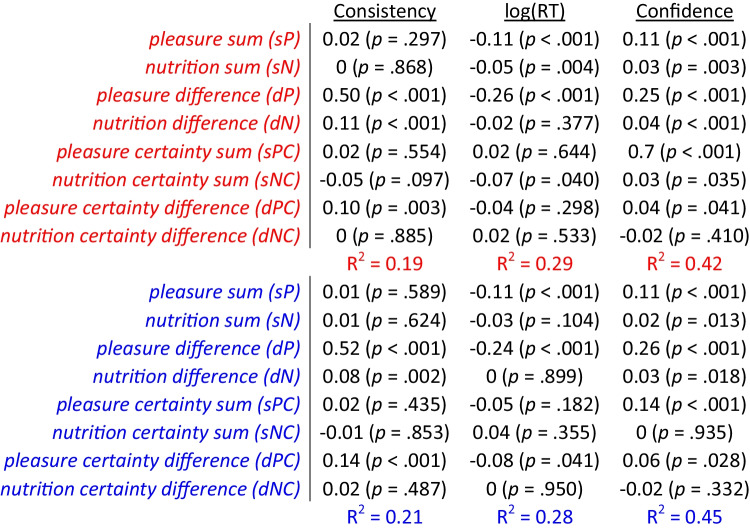
Effects of rating estimates and certainty on choice consistency, response time, and confidence

Table lists the mixed-effects regression coefficients from six separate regressions using either choice consistency, response time, or confidence as the dependent variable, using either pre-choice (in red) or post-choice (in blue) ratings. All regressions included random intercepts and slopes for each variable by participant

**Fig. 7:**
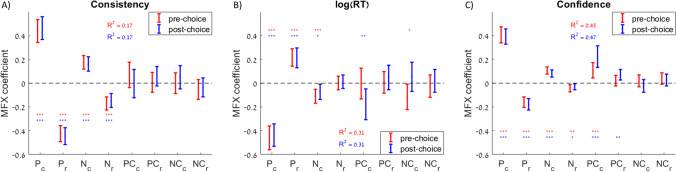
Explanatory power of attribute estimates and certainty on choice, response time, and confidence. Predictive effect of the pleasure, nutrition, pleasure certainty, and nutrition certainty for the chosen option (P_c_, N_c_, PC_c_, NC_c_) and for the rejected option (P_r_, N_r_, PC_r_, NC_r_) on each trial on choice consistency (choosing the option with the higher overall value rating (**A**), response time (**B**), and choice confidence (**C**)). Error bars represent 95% confidence intervals. Significance stars: *p < 0.05; **p < 0.01; ***p < 0.001

We also tested the associations between the same three dependent variables (consistency, RT, and confidence) and the pleasure and nutrition estimate and certainty for the chosen and rejected options. We used three separate regression models to test the effects of all eight independent variables on the three dependent variables (Fig. 7; Table 4). In the regression with choice consistency as the dependent variable, we found significant positive associations with pleasure and nutrition for the chosen option, and significant negative associations with pleasure and nutrition for the rejected option, but no significant associations with any of the certainty terms; the results were similar whether using pre- or post-choice ratings (Fig. 7A). The magnitudes of the weights for chosen options were roughly equal to those for rejected options. When RT was the dependent variable, there were significant negative associations with pleasure and nutrition for the chosen option, and significant positive associations with pleasure (but not nutrition) for the rejected option, and mostly no significant associations with the certainty terms; the results were similar whether using pre- or post-choice ratings (Fig. 7B). The magnitudes of the weights for the chosen options were greater than for the rejected options. When choice confidence was the dependent variable, there were significant positive associations with pleasure and nutrition for the chosen option, and significant negative associations with pleasure and nutrition for the rejected option; these results were similar whether using pre- or post-choice ratings. There were also significant positive associations with pleasure certainty of the chosen option (based on pre- or post-choice ratings) and pleasure certainty of the rejected option (based on post-choice ratings; Fig. 7C). The magnitudes of the weights for the chosen options were greater than for the rejected options.

**Table 4:**
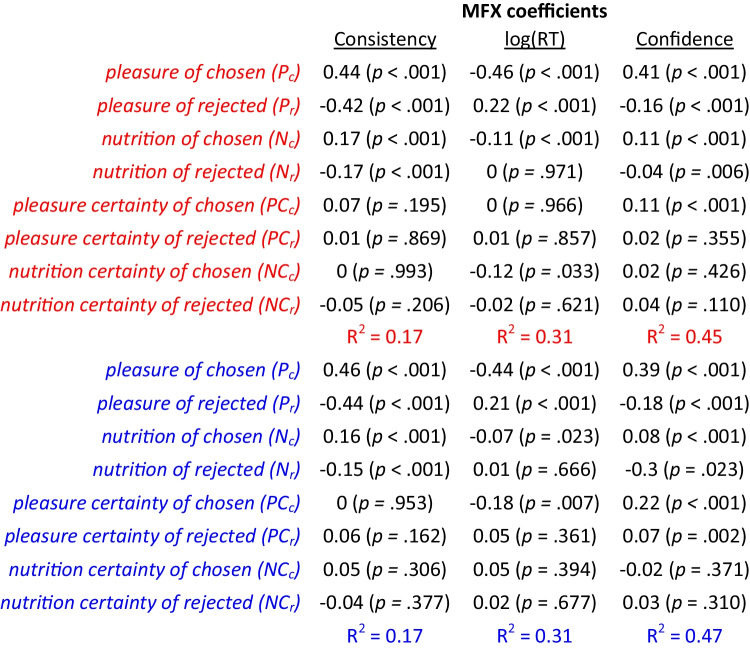
Effects of rating estimates and certainty on choice consistency, response time, and confidence

Table lists the mixed-effects regression coefficients from six separate regressions using either choice consistency, response time, or confidence as the dependent variable, using either pre-choice (in red) or post-choice (in blue) ratings. All regressions included random intercepts and slopes for each variable by participant

#### Choices induce preference refinements

**Fig. 8:**
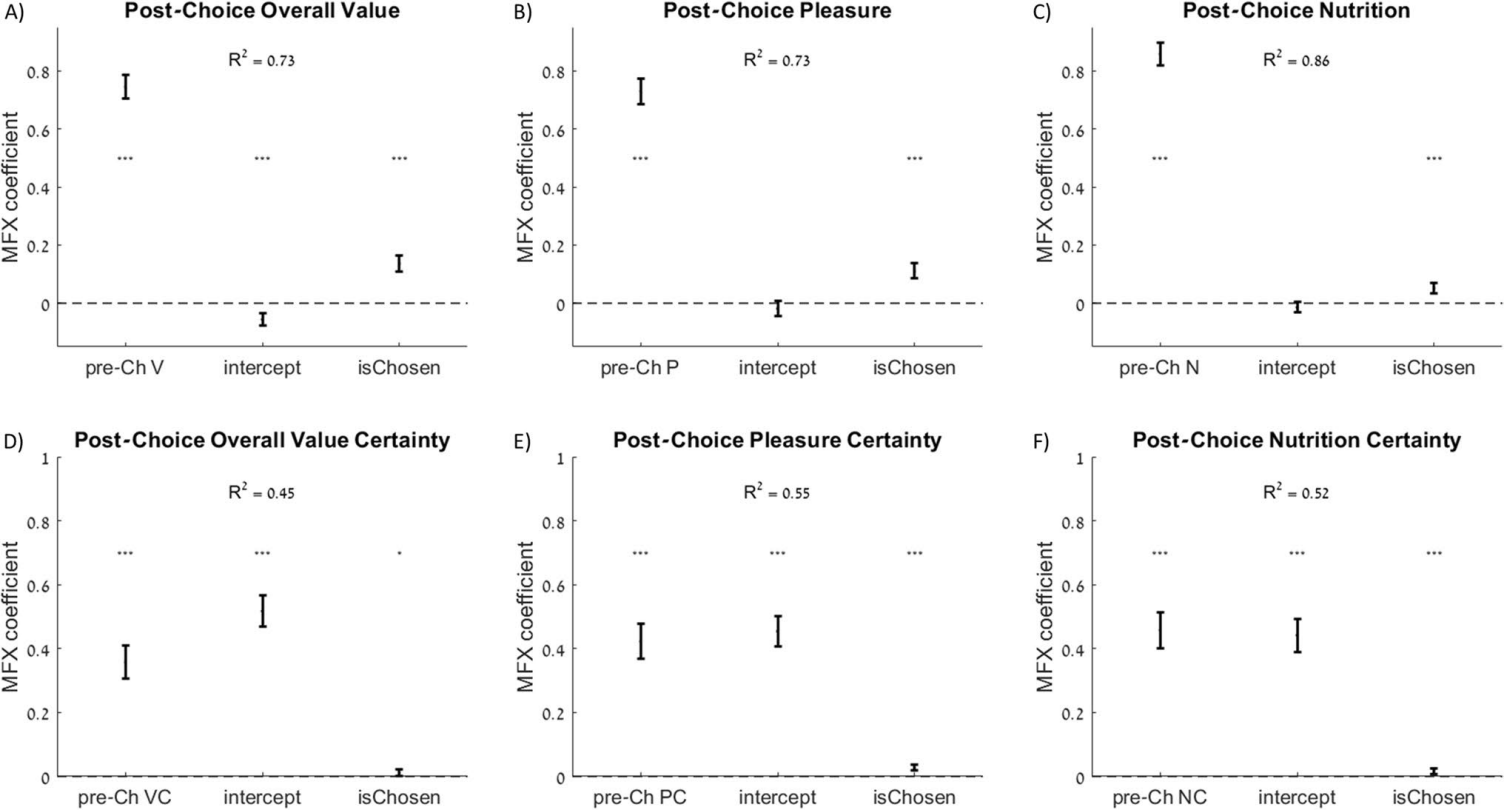
Change in rating estimates and certainty before versus after choices. Rating estimates (top row; V = overall value, P = pleasure, N = nutrition) are highly correlated between first and second rating sessions (pre-choice V, P, and N all have large MFX regression coefficients for post-choice V, P, and N, respectively). The intercept terms have negative coefficients, showing that rejected options tended to decrease in rating estimate. The indicator variables *isChosen* have positive coefficients, showing that chosen options increased in rating relative to rejected options. Rating certainty (bottom row) is also highly correlated between first and second rating sessions. The positive intercept coefficients show that, in contrast to rating estimates, both chosen and rejected options gained in certainty, on average. The positive *isChosen* coefficients show that chosen options increased in certainty more than did rejected options. Error bars represent 95% confidence intervals. Significance stars: *p < 0.05; ***p < 0.001

The purpose of soliciting evaluations both before and after the choice task was to test for choice-induced preference refinements. These preference refinements are thought to occur during the comparison process itself, because decision-makers process information more precisely and/or evaluate additional information beyond the information that they process when required to evaluate options in isolation (Lee and Daunizeau, 2020, 2021). Such preference refinements are typically quantified as the *spreading of alternatives* (SoA) because post-choice ratings of option pairs tend to be further apart relative to their pre-choice ratings. For each choice trial, SoA is defined as the change in overall value rating (from pre- to post-choice) for the option that was chosen minus the change in overall value rating for the other (rejected) option. SoA is traditionally computed as a difference-in-differences metric, calculated as post- minus pre-choice rating for the chosen option minus the post- minus pre-choice rating for the rejected option, which is then averaged across all choices that a participant made. In line with previous work (Izuma et al., 2010, 2015; Izuma and Murayama, 2013; Lee and Coricelli, 2020; Lee and Daunizeau, 2020; Lee and Holyoak, 2021a, 2021b; Sharot et al., 2009, 2010, 2012; Voigt et al., 2017, 2019), we observed a significant spreading of overall value ratings for the chosen relative to rejected options (cross-participant mean of within-participant mean SoA = 0.02, *p* = 0.023). We also observed a significant analogous spreading of ratings of both pleasure (mean = 0.02, *p* = 0.031) and nutrition (mean = 0.02, *p* < 0.001), which replicates recent work (Lee and Holyoak, 2021b). In addition to testing the averaged difference-in-difference scores, we used mixed-effects regressions to test post-choice ratings (separately for overall value, pleasure, and nutrition) against 1) a constant intercept term, 2) pre-choice ratings, and 3) an indicator variable for whether the option was chosen in the choice task. As expected, the pre-choice ratings were strongly correlated with post-choice ratings in all regressions. The coefficients for the intercept terms in the MFX regressions for all three ratings were negative, although this coefficient was only statistically significant in the regression for overall value, and not pleasure or nutrition ratings. In the MFX regression specifications that we used, the intercept term measures the change in ratings for rejected options. Therefore, this result shows that ratings for rejected items were generally lower after choices relative to before, which is in line with previous studies. As expected, in all three MFX regressions, the coefficients for the *chosen* indicator variable were positive and significant, indicating that ratings increased substantially more for items that were chosen relative to those that were rejected (overall value = 0.14, *p* < 0.001; pleasure = 0.11, *p* < 0.001; nutrition = 0.05, *p* < 0.001; Fig. 8A-C).

If the changes in rating estimates that occur between pre- and post-choice ratings (captured by SoA) are indeed due to refinements in option evaluations that occur during choice because of effortful deliberation, we would also expect an increase in rating certainty between the two rating sessions (Lee and Coricelli, 2020; Lee and Daunizeau, 2021). To test for such an effect, we regressed post-choice certainty (separately for overall value, pleasure, and nutrition) on 1) a constant intercept term, 2) pre-choice certainty, and 3) an indicator variable for whether or not the option was chosen in the choice task. We examined the intercept coefficients (i.e., certainty gain for rejected options) and the chosen item indicator coefficients (i.e., *choice-induced certainty gain)*. The coefficients for the intercept terms in all three MFX regressions were positive and significant, indicating that participants felt more certain about their ratings (overall value = 0.52, *p* < 0.001; pleasure = 0.45, *p* < 0.001; nutrition = 0.44, *p* < 0.001; Fig. 8D-F) after having made choices between the options relative to before. In contrast to rating estimates, certainty increased for both chosen and rejected options. Furthermore, all three MFX coefficients for the *isChosen* indicator variable were positive and significant, indicating that participants gained more certainty for options that they chose relative to those that they rejected (overall value = 0.01, *p* = 0.017; pleasure = 0.03, *p* < 0.001; nutrition = 0.02, *p* < 0.001; Fig. 8D-F).

To test whether changes in either value estimates or certainty were systematically greater for the pleasure attribute compared to the nutrition attribute, we performed an additional MFX regression of post-choice attribute ratings (certainty) on pre-choice attribute ratings (certainty), an intercept term, an indicator variable (isP) to designate whether the rating (certainty) was for pleasure, an indicator variable (isChosen) to designate whether the option was chosen, and the interaction of isChosen and isP. We found that the pleasure ratings for chosen options increased significantly more than did the nutrition ratings (MFX coefficient for isChosen*isP = 0.06, *p* < 0.001) and the pleasure certainty for chosen options tended to increase significantly more than did the nutrition certainty (MFX coefficient for isChosen*isP = 0.01, *p* = 0.056).

The amount of spreading of ratings between chosen and rejected alternatives is related to differences in pre-choice ratings and the sum of the pre-choice certainty across option pairs. For example, previous work reported negative associations between SoA, in terms of overall value, and overall value difference and overall value certainty sum (Lee and Coricelli, 2020; Lee and Daunizeau, 2020, 2021), as well as a positive association between SoA and attribute disparity (Lee and Holyoak, 2021b). Our data replicated most of these findings (see Supplementary Material) and also showed similar patterns with respect to a novel variable that we call the spreading of alternatives’ certainty (*SoAC*, calculated in a manner analogous to SoA). Recent work has shown similar associations for SoA in terms of pleasure and nutrition attributes (Lee and Holyoak, 2021b). Our data also replicated these findings (see Supplementary Material).

#### Choice-induced preference refinements facilitate choice

**Fig. 9:**
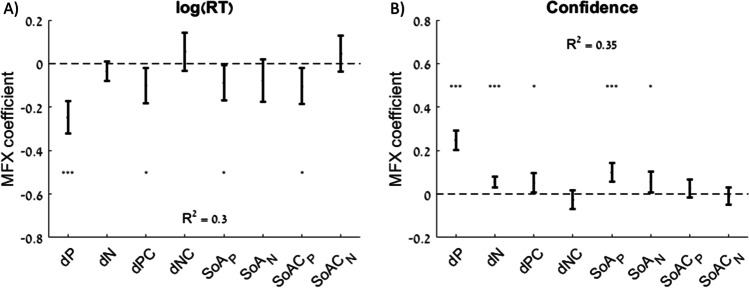
Explanatory power of attribute estimates and certainty, spreading of alternatives, and spreading of alternatives’ certainty on response time and confidence. Predictive effect of the difference in pleasure (dP), difference in nutrition (dN), difference in pleasure certainty (dPC), difference in nutrition certainty (dNC), spreading of alternatives with respect to pleasure (SoA_P_), spreading of alternatives with respect to nutrition (SoA_N_), spreading of alternatives’ certainty with respect to pleasure (SoAC_P_), and spreading of alternatives’ certainty with respect to nutrition (SoAC_N_) of the options on each trial on response time (**A**) and choice confidence (**B**). Error bars represent 95% confidence intervals. Significance stars: *p < 0.05; ***p < 0.001

Previous work postulated that the SoA phenomenon is a cognitive process that occurs during choice deliberation (Lee and Daunizeau, 2020, 2021; Schonberg and Katz, 2020; Voigt et al., 2019) or potentially any other situation in which additional information is processed about the available options (Lee and Holyoak, 2021b). Specifically, the “spreading of alternatives” should result in a decision becoming easier as the overall value estimates of the choice options become more distinct. According to this theory, SoA occurs during the choice task even though it is calculated by the experimenter using ratings obtained before and after the choice task. Previous work has shown that SoA that occurs during the choice task remains observable in post-choice ratings (Lee and Holyoak, 2021a). It thus follows that choice variables impacted by overall value difference (RT and confidence^1^) should be associated with SoA in a manner that suggests the choice has become easier (i.e., faster RT and higher confidence). This result has been reported in previous studies (Lee and Coricelli, 2020; Lee and Daunizeau, 2020, 2021; Lee and Holyoak, 2021a, 2021b). We replicated the previous findings with respect to SoA for overall value and observed similar results with respect to SoAC (see Supplementary Material). We also tested for associations between SoA_P_ and SoA_N_ and RT or confidence. Specifically, we regressed RT and confidence (separately) on the differences in pleasure, nutrition, pleasure certainty, and nutrition certainty, as well as on SoA_P_, SoA_N_, SoAC_P_, and SoAC_N_ (Fig. 9; Table 5). For RT, all variables related to pleasure showed a negative association, but none of the associations with nutrition-related variables were significant. For confidence, pleasure and nutrition difference both had positive associations, as did pleasure certainty difference and both SoA_P_ and SoA_N_.

**Table 5:** Effects of rating estimates, certainty, and spreading of alternatives on response time and confidence

	log(RT)	Confidence
*pleasure difference (dP)*	−0.25 (*p* < 0.001)	0.25 (*p* < 0.001)
*nutrition difference (dN)*	−0.03 (*p* = 0.129)	0.06 (*p* < 0.001)
*pleasure certainty difference (dPC)*	−0.10 (*p* = 0.016)	0.05 (*p* = 0.020)
*nutrition certainty difference (dNC)*	0.06 (*p* = 0.213)	−0.03 (*p* = 0.225)
*spreading of alternatives: pleasure (SoA*_*P*_*)*	−0.09 (*p* = 0.034)	0.10 (*p* < 0.001)
*spreading of alternatives: nutrition (SoA*_*N*_*)*	−0.08 (*p* = 0.114)	0.06 (*p* = 0.021)
*spreading of alternatives’ certainty: pleasure (SoAC*_*P*_*)*	−0.10 (*p* = 0.013)	0.03 (*p* = 0.219)
*spreading of alternatives’ certainty: nutrition (SoAC*_*N*_*)*	0.05 (*p* = 0.269)	−0.01 (*p* = 0.632)
	R^2^ = 0.30	R^2^ = 0.35

Table lists the mixed-effects regression coefficients from two separate regressions using either response time or confidence as the dependent variable. All regressions included random intercepts and slopes for each variable by participant

### Discussion

This second experiment indicates that the act of making a choice leads to refinements in evaluations (both estimates and certainty) of options with respect to specific attributes as well as overall value. Comparing the pre- and post-choice ratings allowed us to determine how the various effects that we examined in Experiment 1 differed after participants had made choices between pairs of options. We found that across all eight regression models that we tested using either pre- or post-choice ratings, all but one yielded greater explanatory power (adjusted R^2^) when including post-choice ratings. This supports the hypothesis that decision-makers refine their assessments of value during choice deliberation, so that they are more (subjectively) accurate and precise after choices than they were before (Lee and Daunizeau, 2020, 2021).

Contrasting post- versus pre-choice ratings, and their associations with choice behavior, revealed choice-induced preference refinements. These refinements were quantified as the *spreading of alternatives* (SoA), where the differences in value estimates (overall, pleasure, and nutrition) for the chosen option versus the rejected option were significantly larger after the choices relative to before, in line with previous work (Izuma et al., 2010; Lee and Coricelli, 2020; Lee and Daunizeau, 2020, 2021; Lee and Holyoak, 2021a, 2021b; Voigt et al., 2019). We also showed the novel result that choices induced an asymmetrical gain in rating certainty (overall value, pleasure, and nutrition). Our results indicate that both the subjective sense of attributes and overall value as well as the subjective sense of certainty about those ratings are refined during choice deliberation. Options that are ultimately chosen appear to undergo more refinement than rejected options. Interestingly, the pleasure attribute seemed to undergo greater refinement than the nutrition attribute, in line with the greater weights for pleasure relative to nutrition in all the rating and choice regression analyses that we conducted. Furthermore, both types of refinement (SoA and SoAC) are associated with reduced response times and increased choice confidence, indicating that the process that leads to refinement also is instrumental to choice. Finally, beyond the increase in certainty about the value of the chosen option relative to the rejected one, we found a clear gain in certainty (on average) across all options. This replicates previous work (Lee and Coricelli, 2020; Lee and Daunizeau, 2021) and suggests that the observed changes in value estimates are more likely due to true preference refinements rather than simply random fluctuations in the estimates.

## General discussion

We examined how subjective feelings of certainty about subjective assessments of individual decision-relevant option attributes contribute to key aspects of choice behavior: the consistency between choices and isolated ratings of value, deliberation time during a decision, and reports of confidence about having chosen the preferred options. Previous work has demonstrated that people possess the metacognitive ability to assess their certainty in their value estimates for choice options, and that levels of certainty influence decision and learning behaviors (Cortese, 2022; Cortese et al., 2020; De Martino et al., 2013; Gwinn and Krajbich, 2020; Lee and Coricelli, 2020; Lee and Daunizeau, 2020, 2021; Polanía et al., 2019). Thus far, studies of metacognition in judgement and decision-making have focused on the overall value or percept of a stimulus. We expand previous findings in two main ways: First, in both of our experiments, we show that the influence of the certainty about an attribute on evaluations of and decisions between snack foods is proportional to the influence of the value of that attribute on the evaluation or decision. In particular, across participants, the associations of pleasure-related variables with all dependent variables related to either overall value or choice were greater than the associations of nutrition-related variables. Thus, metacognitive assessments of value certainty or choice confidence are not equally weighted assessments of the total amount of (un)certainty across all attributes and options. Instead, our results suggest that metacognitive computations are influenced by unequally weighted combinations of information about the different attributes. In this way, metacognition is similar to computations of overall value and choices themselves, which are known to be influenced by subjectively weighed combinations of attribute estimates.

Second, in Experiment 2, we demonstrate that metacognitive evaluations of both overall value and individual attributes are refined during choice deliberation. Previous work has shown that value estimates systematically change from pre- to post-choice rating sessions (Izuma et al., 2010, 2015; Izuma and Murayama, 2013; Lee and Coricelli, 2020; Sharot et al., 2009, 2010, 2012; Voigt et al., 2017, 2019) and that post-choice estimates are more closely aligned with choice behavior (Lee and Daunizeau, 2020, 2021; Lee and Holyoak, 2021a, 2021b; Lee and Pezzulo, 2022). We replicate these findings at the level of both overall value and individual attributes. We also demonstrate similar effects with respect to value certainty. Previous work showed that certainty about overall value increases with choices or additional rating sessions (Lee and Coricelli, 2020; Lee and Daunizeau, 2021). We show that certainty also increases at the level of individual attributes but that these increases are greater for attributes that had a greater impact on the decisions (i.e., pleasure > nutrition). Gains in certainty are also related to the choice outcome: chosen options show a greater increase in certainty than do rejected options. Furthermore, we show that spreading of alternatives with respect to value and/or certainty reduces response time and increases choice confidence, suggesting that the evaluation refinements are instrumental to choice. Together, these findings support the hypothesis that subjectively weighted combinations of individual attributes (both estimates and certainty) directly contribute to both cognitive *and* metacognitive processes in value-based decision-making.

Metacognition is thought to aid decision-makers in choosing the best option from among alternatives (Desender et al., 2021; Lee, Daunizeau, et al., 2022b; Lee and Daunizeau, 2021; van den Berg et al., 2016). It also is believed that metacognition about choices that have already been made can be beneficial for future decisions of a similar nature (Fleming and Daw, 2017; Yeung and Summerfield, 2012). More generally, the ability to monitor one’s own performance is thought to be important for efficient learning (Cortese, 2022; Cortese et al., 2020). It has been shown that people can unconsciously learn the relative importance of different decision features or attributes via a reinforcement learning process modulated by choice confidence (Cortese et al., 2020). While that previous work operationalized the multiattribute nature of the decision as abstract multidimensional patterns of brain activity, the finding may hold for explicit attribute dimensions such as the pleasure and nutrition of snack foods as well. Pleasure and nutrition (or “tastiness” and “healthiness”) have previously been decoded from brain activity using multivariate pattern analysis, even when participants were not instructed to explicitly consider those attributes (Schubert et al., 2021; Suzuki et al., 2017). It could be that choice confidence levels are both informed by such latent neural representations of option attributes and that the relative contribution of the value certainty for each attribute matches the relative contribution of the value estimates for each attribute, because both types of information originate in the same neural representations.

When interpreted in the light of the previous findings on metacognition and learning mentioned above, our current results suggest that repeatedly choosing between options with a specific ordering of attribute importance would reinforce that ordering and one’s confidence in choices that align with it. Recall that confidence increased more in the more choice-relevant attribute, pleasure, relative to the less important attribute, nutrition. This type of internal feedback loop would benefit future choice behavior if it were based on an appropriate ranking of attribute importance (i.e., if attribute ranks align with the context and current goal). However, if the context or goal were to change, having confidence constructed in proportion to a now suboptimal ranking of attribute importance would instead impair efficient learning and behavior. Thus, it will be important for future studies to examine how readily confidence judgements can be adapted to match changes in goals or environments.

Neuroimaging studies have revealed both domain-specific and domain-general representations of attributes and overall values as well as decision confidence. Many brain regions show domain-specific correlations with the values of specific types of rewards (Chib et al., 2009). On the other hand, the medial prefrontal cortex (mPFC), striatum, and posterior cingulate cortex have been shown to correlate with the overall value of a wide range of goods (Bartra et al., 2013; Chib et al., 2009; Clithero and Rangel, 2013; Levy and Glimcher, 2012), and overlapping voxels in mPFC have also been shown to correlate with ratings for distinct individual attributes (Hare et al., 2009, 2011). In contrast, lateral prefrontal cortex and amygdala have been found to correlate only with a subset of attributes (Hare et al., 2011; Maier et al., 2015). In terms of metacognitive representations, activity in the mPFC also correlates with unidimensional confidence ratings in value-based choice paradigms (De Martino et al., 2013; Folke et al., 2016) and shows a domain-general association with confidence across perceptual judgements and memory tasks (Molenberghs et al., 2016; Morales et al., 2018). However, lateral prefrontal regions once again show domain-specific associations with either perception or memory (Morales et al., 2018). Thus, it may be that the certainties about individual attributes within the same option are represented in either overlapping or distinct circuits within the brain. This will be an important question to address in future studies.

In this study, we conceptualized “pleasure” as an attribute that directly contributes information related to overall value estimation as well as value-based decisions. However, one could argue that pleasure might instead describe the overall impression that a decision-maker forms about each option while thinking about them. In the latter case, the pleasure and overall value ratings would be redundant and thus highly correlated, which might confound our results. In our experimental instructions, we attempted to forestall such a confound by explicitly describing pleasure as an attribute, not a feeling of affect. In our data, the correlations between pleasure ratings and overall value ratings (across participants) are 0.65 in Experiment 1 and 0.62 and 0.66 (pre- and post-choice) in Experiment 2 (see Supplementary Material for matrices of correlations between all variables). Thus, pleasure is a substantial component of overall value, but it explains less than half of the variance in overall values. Moreover, if pleasure and overall value were somehow capturing the same affective state, we would expect to find similar correlations between nutrition and either pleasure or overall value. Instead, we find that the correlations between pleasure and nutrition (0.05 in Experiment 1 and −0.09 and 0.05 in Experiment 2) are significantly different (*p* < 0.001) from the correlations between overall value and nutrition are positive (0.17 in Experiment 1 and 0.17 and 0.26 in Experiment 2). These data demonstrate that pleasure and overall value are not measuring the same subjective impressions in our experiments.

There are some limitations of the current work that should be addressed in future studies. First, we examined choices over snack food items, because foods are familiar, multiattribute goods that humans evaluate and make choices between on a regular basis. However, future work should test whether our findings extend to other types of goods or experiences. Second, we had participants rate and report confidence for only two separate attributes. Tests that use more attributes are necessary to examine further the relationship between attributes’ influence on evaluations of overall value, choices, and certainty or confidence. Lastly, participants did not actually receive the snack foods that they chose in this study, which might have reduced motivation to rate and choose (subjectively) accurately. The high correlations and consistency between rating types and choices in our data set indicate that participants generally rated and chose carefully. Nevertheless, studies using incentivized ratings and choices may provide additional motivation, and consequently, more sensitivity to detect subtle behavioral effects.

## Conclusions

We have provided strong initial evidence that just like first-order (cognitive) evaluations of multiattribute choice options, second-order assessments of decision confidence are subjective and more influenced by highly choice-relevant attributes compared with attributes that are only marginally important in determining decision outcomes. These findings provide a basis and impetus for additional investigation into the nature of multiattribute confidence and its implications for beneficial and detrimental forms of cognition and behavior.

## Supplementary Information

ESM 1 (DOCX 1360 kb)

## Footnotes

1 We do not include consistency, because some choices can be classified as changes of mind (i.e., dV_1_ and dV_2_ would have opposite signs). These cannot be classified as errors, however, as value refinements might have caused decision-makers to update their preferences on these trials. For such trials, choice consistency would be anticorrelated with SoA.
