# Supplementary material for: Value certainty and choice confidence are multidimensional constructs that guide decision-making
Source: Cogn Affect Behav Neurosci. 2023 Jan 11;23(3):503–21. doi: 10.3758/s13415-022-01054-4 (PMC10390628; doi:10.3758/s13415-022-01054-4)
Supplement: Supplementary file 1 — (DOCX 1360 kb) [file 13415_2022_1054_MOESM1_ESM.docx]

**Supplementary Material**

for

**Value certainty and choice confidence are multidimensional constructs that guide decision-making**

Douglas G. Lee ^1,2^ and Todd A. Hare ^2,3^

^1^ Tel Aviv University, School of Psychological Sciences

^2^ Zurich Center for Neuroeconomics, Department of Economics, University of Zurich

^3^ Neuroscience Center Zurich, University of Zurich and ETH Zurich

1. **Supplementary Results.**

*1.1 Value certainty correlates with value estimate and estimate extremity*

Previous studies have shown that feelings of certainty about overall value estimates correlate with the overall value estimates themselves (Lebreton et al., 2015; Lee & Coricelli, 2020). Specifically, reported levels of certainty about overall value are higher when the overall value estimates are higher, as well as when they are farther away from the point of neutrality (in either direction). To test this association in our data, we regressed overall value certainty on overall value and overall value extremity (|overall value|), separately for data from Experiments 1 an 2. For both datasets, both MFX coefficients were significantly greater than zero (**Exp1**: overall value: 0.08, *p* < .001; overall value extremity: 0.42, *p* < .001; adjusted R^2^ = 0.46; **Exp2**: overall value: 0.07, *p* < .001; overall value extremity: 0.46, *p* < .001; adjusted R^2^ = 0.47). For Experiment 2, we repeated the same regression analysis, this time using post-choice ratings. Post-choice ratings yielded MFX coefficients that were similar to those for pre-choice ratings, and they also explained more of the variance (overall value: 0.06, *p* < .001; overall value extremity: 0.50, *p* < .001; adjusted R^2^ = 0.54). We then tested whether the same pattern holds at the level of individual attributes by regressing pleasure certainty on pleasure and pleasure extremity, and nutrition certainty on nutrition and nutrition extremity. All MFX coefficients were significantly greater than zero (**Exp1**: pleasure: 0.08, *p* < .001; pleasure extremity: 0.42, *p* < .001; adjusted R^2^ = 0.48; nutrition: 0.05, *p* < .001; nutrition extremity: 0.61, *p* < .001; adjusted R^2^ = 0.56; **Exp2**: pleasure: 0.09, *p* < .001; pleasure extremity: 0.46, *p* < .001; adjusted R^2^ = 0.50; nutrition: 0.04, *p* < .001; nutrition extremity: 0.68, *p* < .001; adjusted R^2^ = 0.52). For Experiment 2, we repeated the same regression analysis, this time using post-choice ratings. Post-choice ratings yielded MFX coefficients that were similar to those for pre-choice ratings, and they also explained more of the variance (pleasure: 0.08, *p* < .001; pleasure extremity: 0.45, *p* < .001; adjusted R^2^ = 0.53; nutrition: 0.04, *p* < .001; nutrition extremity: 0.64, *p* < .001; adjusted R^2^ = 0.55).

*1.2 Attribute estimates and certainty explain overall value estimates*

To tie together the findings reported above, we tested how well overall value estimates could be explained by a combination of attribute estimates and attribute certainty. Specifically, we regressed overall value on pleasure, nutrition, pleasure certainty, and nutrition certainty. For both experiments, the MFX coefficients for pleasure, nutrition, and pleasure certainty were positive and significant, but the MFX coefficient for nutrition certainty was not statistically significant (**Exp1**: pleasure: 0.76, *p* < .001; nutrition: 0.14, *p* < .001; pleasure certainty: 0.06, *p* < .001; nutrition certainty: 0.01, *p* = .669; adjusted R^2^ = 0.69; **Exp2**: pleasure: 0.71, *p* < .001; nutrition: 0.20, *p* < .001; pleasure certainty: 0.05, *p* = .006; nutrition certainty: 0.02, *p* = .241; adjusted R^2^ = 0.67). For Experiment 2, we repeated the same regression analysis, this time using post-choice ratings. Post-choice ratings yielded MFX coefficients that were similar to those for pre-choice ratings, and they also explained more of the variance (pleasure: 0.74, *p* < .001; nutrition: 0.24, *p* < .001; pleasure certainty: 0.03, *p* = .027; nutrition certainty: 0, *p* = .893; adjusted R^2^ = 0.76).

*1.3 Attribute congruence influences overall value certainty*

We hypothesized that one factor that should support subjective certainty about the overall value of an option would be the congruence of its attributes. For example, an option with a very high rating for one attribute but a very low rating for another attribute might have a medium overall value estimate with low certainty. An option with moderate ratings for both attributes (i.e., congruent attribute ratings) might also have a medium overall value estimate, but with higher certainty. To test this hypothesis, we regressed overall value certainty on pleasure certainty, nutrition certainty, and *attribute congruence*. We defined attribute congruence as the interaction between the pleasure and nutrition ratings for each item (so congruence is greatest when both ratings are -1 or both ratings are 1, least when one rating is -1 and the other is 1). All MFX coefficients were positive and significant (**Exp1**: pleasure certainty: 0.30, *p* < .001; nutrition certainty: 0.12, *p* < .001; attribute congruence: 0.03, *p* < .001; adjusted R^2^ = 0.46; **Exp2**: pleasure certainty: 0.31, *p* < .001; nutrition certainty: 0.11, *p* < .001; attribute congruence: 0.03, *p* < .001; adjusted R^2^ = 0.43; Figure S1). For Experiment 2, we repeated the same regression analysis, this time using post-choice ratings. Post-choice ratings yielded MFX coefficients that were similar to those for pre-choice ratings, and they also explained more of the variance (pleasure certainty: 0.37, *p* < .001; nutrition certainty: 0.16, *p* < .001; attribute congruence: 0.05, *p* < .001; adjusted R^2^ = 0.54; Figure S1).


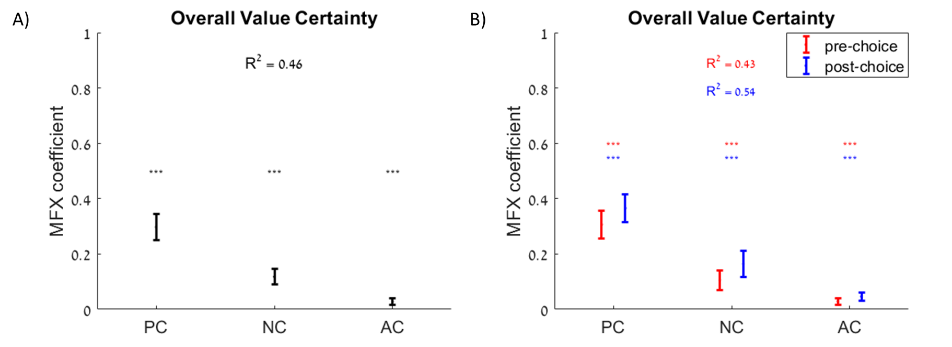


**Figure S1:** **Explanatory power of attribute certainty and congruence to overall value certainty.** Beyond the explanatory power of pleasure certainty (PC) and nutrition certainty (NC) for overall value certainty (VC), the congruence of the attributes (AC, within options) also has a positive association with VC. Error bars represent 95% confidence intervals. Significance stars: *** *p* < .001.

*1.4 Choice behavior can be explained by value estimates and certainty plus attribute disparity*

We then tested whether our current data replicate previous findings reported in the literature regarding the relationships between three dependent variables 1) choice consistency (choosing the option with the higher overall value rating), 2) response time (RT), and 3) choice confidence, and five previously-established independent variables. These five independent variables are 1) the difference and 2) sum of overall value estimates, 3) the difference and 4) sum of overall value certainty, and 5) attribute disparity. Many studies have shown that the difference in overall value estimates (defined as higher-valued option minus lower-valued option) across options in a choice pair has a positive association with consistency and confidence and a negative association with RT, and similar associations have also been shown with respect to the sum of overall value estimates (Lee & Usher, 2021; Shevlin et al., 2022; Smith & Krajbich, 2019). Furthermore, the sum of overall value certainty and the difference in overall value certainty (defined as certainty of the option with higher overall value minus the certainty of the option with lower overall value) have been shown to have positive associations with consistency and confidence, and negative associations with RT (Lee & Coricelli, 2020; Lee & Daunizeau, 2020, 2021)). Finally, it has been shown that attribute disparity correlates positively with choice consistency and confidence, and negatively with RT (Lee & Holyoak, 2021b). Attribute disparity accounts for the fact that choice options could be composed of very different attribute estimates, even if they were rated with similar overall values. We calculated disparity as:

${disparity}_{i,j}\triangleq\left| \frac{\left[ P_{i} N_{i} \right]*\left[ \begin{matrix} {-b}_{P} \\ b_{N} \end{matrix} \right]-\left[ P_{j} N_{j} \right]*\left[ \begin{matrix} -b_{P} \\ b_{N} \end{matrix} \right]}{\left\| \begin{matrix} -b_{P} \\ b_{N} \end{matrix} \right\|} \right|, for options i,j.$ (1)

where pleasure and nutrition are the pleasure and nutrition ratings for the specific options being compared, and b_P_ and b_N_ are the GLM coefficients from a regression of overall value on pleasure and nutrition (within participants). Using this measure of disparity, one can transform the space representing choice options from attribute space (i.e., a two-dimensional space composed of pleasure and nutrition axes) to a new two-dimensional space in which one dimension is overall value difference and the other is disparity (Lee & Holyoak, 2021b).

We used three separate regression models to test for the effects of all five of these independent variables on each of the three choice-related dependent variables in our data. For the dependent variable choice consistency, we replicated the associations with overall value difference in both experiments, and the associations with overall value certainty difference and disparity in Experiment 1 only (Figure S2, Table S1). When using choice RT as the dependent variable, we replicated the associations with disparity and overall value sum and difference in both experiments, and the sum of overall value certainty in Experiment 2 only (Figure S2, Table S1). Lastly, when using choice confidence as the dependent variable, we found a trend for overall value certainty difference in Experiment 1, and replicated all other associations in Experiment 1 and all associations in Experiment 2 (Figure S2, Table S1). Note that, for Experiment 2, post-choice ratings yielded MFX coefficients that were similar to those for pre-choice ratings, but now more variables showed significant associations and the overall percentage of variance explained was greater.


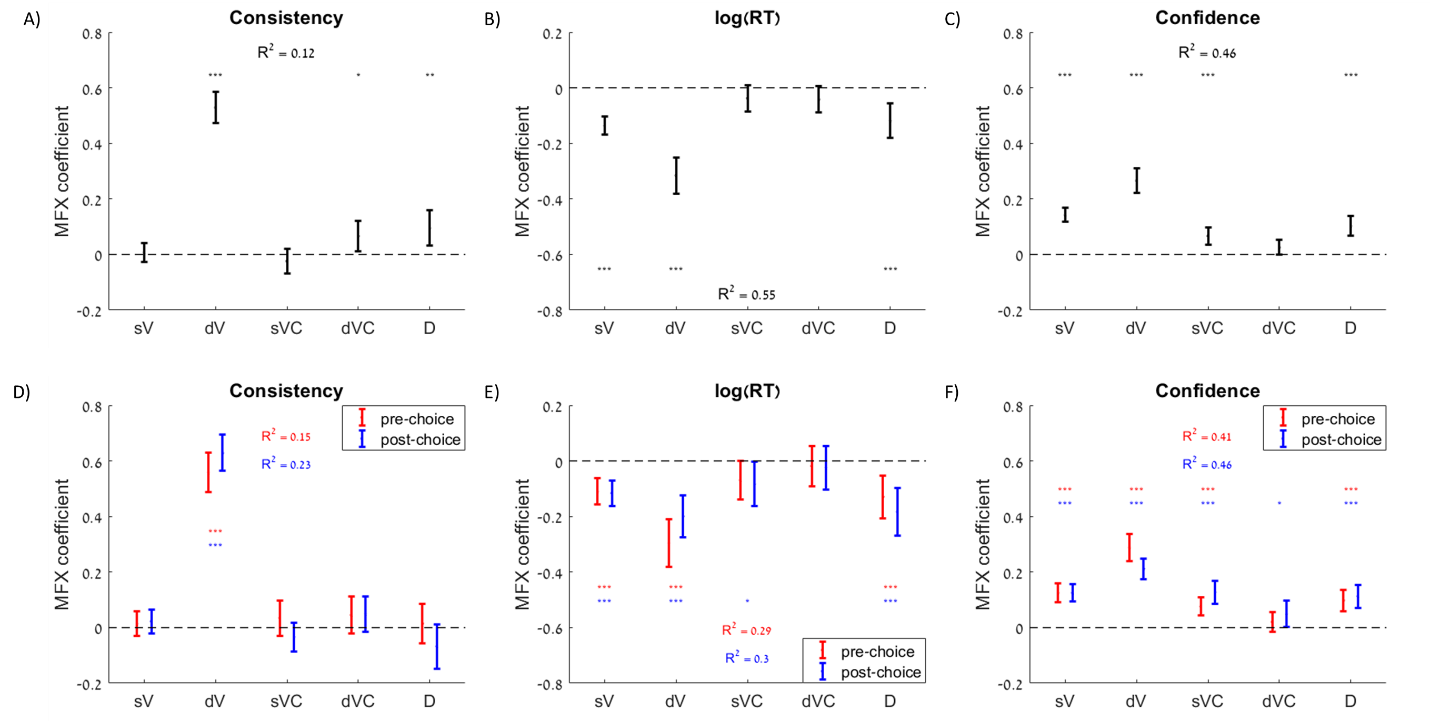


**Figure S2: Explanatory power of value estimates and certainty on choice, response time, and confidence.** Predictive effect of the sum of overall value (sV), difference in overall value (dV), sum of overall value certainty (sVC), difference in overall value certainty (dVC), and attribute disparity (D) of the options on each trial on choice consistency (choosing the option with the higher overall value rating; left column), response time (center column), and choice confidence (right column). Top row shows results for Experiment 1, bottom row shows results for Experiment 2. Error bars represent 95% confidence intervals. Significance stars: * *p* < .05, ** *p* < .01, *** *p* < .001.

**Table S1.** Effects of rating estimates and certainty and attribute disparity on choice consistency, response time, and confidence.

|  | Consistency | log(RT) | Confidence |
| --- | --- | --- | --- |
| *Overall value sum (sV)* | 0.01 (*p* = .658) | -0.14 (*p* < .001) | 0.14 (*p* < .001) |
| *Overall value difference (dV)* | 0.53 (*p* < .001) | -0.32 (*p* < .001) | 0.27 (p < .001) |
| *Overall value certainty sum (sVC)* | -0.02 (*p* = .303) | -0.04 (*p* = .116) | 0.07 (*p* < .001) |
| *Overall value certainty difference (dVC)* | 0.07 (*p* = .018) | -0.04 (*p* = .080) | 0.03 (*p* = .060) |
| *Attribute disparity (D)* | 0.10 (*p* = .003) | -0.12 (*p* < .001) | 0.10 (p < .001) |
|  | R^2^ = 0.12 | R^2^ = 0.55 | R^2^ = 0.46 |
| *Overall value sum (sV)* | 0.01 (*p* = .538) | -0.11 (*p* < .001) | 0.13 (*p* < .001) |
| *Overall value difference (dV)* | 0.56 (*p* < .001) | -0.30 (*p* < .001) | 0.29 (*p* < .001) |
| *Overall value certainty sum (sVC)* | 0.03 (*p* = .285) | -0.07 (*p* = .052) | 0.08 (*p* < .001) |
| *Overall value certainty difference (dVC)* | 0.05 (*p* = .188) | -0.02 (*p* = .618) | 0.02 (*p* = .270) |
| *Attribute disparity (D)* | 0.01 (*p* = .678) | -0.13 (*p* < .001) | 0.10 (*p* < .001) |
|  | R^2^ = 0.15 | R^2^ = 0.29 | R^2^ = 0.41 |
| *Overall value sum (sV)* | 0.02 (*p* = .302) | -0.12 (*p* < .001) | 0.13 (*p* < .001) |
| *Overall value difference (dV)* | 0.63 (*p* < .001) | -0.20 (*p* < .001) | 0.21 (*p* < .001) |
| *Overall value certainty sum (sVC)* | -0.03 (*p* = .209) | -0.08 (*p* = .042) | 0.13 (*p* < .001) |
| *Overall value certainty difference (dVC)* | 0.05 (*p* = .133) | -0.02 (*p* = .549) | 0.05 (*p* = .038) |
| *Attribute disparity (D)* | -0.07 (*p* = .098) | -0.18 (*p* < .001) | 0.11 (*p* < .001) |
|  | R^2^ = 0.23 | R^2^ = 0.30 | R^2^ = 0.46 |

This table lists the mixed-effects regression coefficients from nine separate regressions using either choice consistency, response time, or confidence as the dependent variable, for Experiment 1 (in black), Experiment 2 using pre-choice ratings (in red), and Experiment 2 using post-choice ratings (in blue). All regressions included random intercepts and slopes for each variable by participant.

*1.5 Choice-induced preference refinements*

In the data from Experiment 2, we tested whether spreading of alternatives’ estimates (SoA) and the spreading of alternatives with respect to certainty (*SoAC*, calculated in a manner analogous to SoA) were associated with the five independent variables that we examined above: sum and difference in overall value estimates, sum and difference in overall value certainty, and attribute disparity, when those variables were defined using pre-choice ratings. Previous studies reported negative associations between SoA and overall value difference and overall value certainty sum (Lee & Coricelli, 2020; Lee & Daunizeau, 2020, 2021), as well as a positive association between SoA and attribute disparity (Lee & Holyoak, 2021b); tests of the other variables have not been reported previously. We regressed SoA and SoAC (separately) on these five independent variables. When testing the spreading of alternatives’ estimates, we replicated the associations with overall value difference and disparity, but did not find any significant associations with the other variables (Figure S3A; Table S2). When testing the spreading of alternatives’ certainty, we found positive associations with overall value sum and disparity, and a negative association with overall value certainty difference (Figure S3B; Table S2).


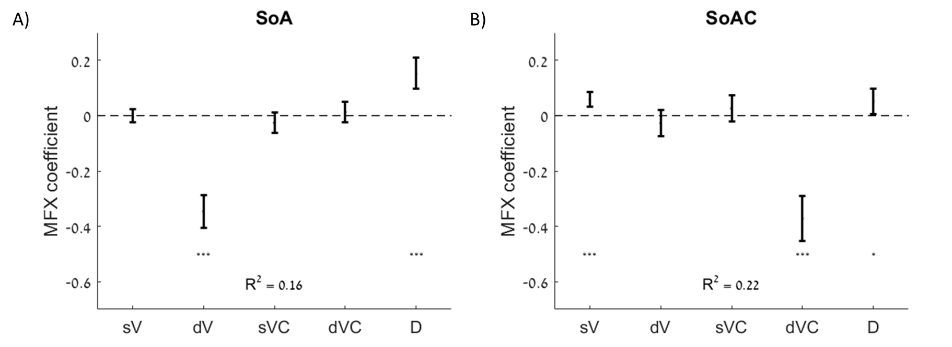


**Figure S3: Explanatory power of value estimates and certainty on the spreading of alternatives.** Predictive effect of the sum of overall value (sV), difference in overall value (dV), sum of overall value certainty (sVC), difference in overall value certainty (dVC), and attribute disparity (D) of the options on each trial on the spreading of alternatives with respect to overall value estimates (SoA; A) and overall value certainty (SoAC; B). Error bars represent 95% confidence intervals. Significance stars: * *p* < .05, *** *p* < .001.

**Table S2.** Effects of rating estimates and certainty and attribute disparity on the spreading of alternatives’ estimates and certainty.

|  | Spreading of Alternatives (SoA) | Spreading of Alternatives’ Certainty (SoAC) |
| --- | --- | --- |
| *Overall value sum (sV)* | 0 (*p* = .901) | 0.06 (*p* < .001) |
| *Overall value difference (dV)* | -0.35 (*p* < .001) | -0.03 (*p* = .249) |
| *Overall value certainty sum (sVC)* | -0.03 (*p* = .167) | 0.03 (*p* = .274) |
| *Overall value certainty diff. (dVC)* | 0.01 (*p* = .492) | -0.37 (*p* < .001) |
| *Attribute disparity (D)* | 0.15 (*p* < .001) | 0.05 (*p* = .025) |
|  | R^2^ = 0.16 | R^2^ = 0.22 |

This table lists the mixed-effects regression coefficients from two separate regressions using either the spreading of alternatives or the spreading of alternatives’ certainty as the dependent variable. All regressions included random intercepts and slopes for each variable by participant.

We next tested whether our data replicate the previously-reported associations between pleasure difference and SoA in terms of pleasure ratings (SoA_P_), and between nutrition difference and SoA in terms of nutrition ratings (SoA_N_). We also included novel tests for associations between pleasure sum, pleasure certainty sum, and pleasure certainty difference and the spreading of alternatives in terms of pleasure certainty (SoAC_P_), and analogous regressions for nutrition certainty (SoAC_N_). We regressed each SoA term on the four related independent variables (i.e., pleasure-related variables in the same regressions, nutrition-related variables in the same regressions). For SoA_P_ and SoA_N_, we replicated the associations with pleasure difference (MFX coefficient = -0.21, *p* < .001) and nutrition difference (MFX coefficient = -0.05, *p* < .001), respectively, but did not find any significant associations between attribute certainty and the spreading of attribute ratings. For SoAC_P_ and SoAC_N_, we found the expected negative associations with pleasure certainty difference and nutrition certainty difference, repsectively (MFX coefficient for SoAC_P_ = -0.34, *p* < .001; MFX coefficient for SoAC_N_ = -0.29, *p* < .001).

Finally, we checked whether preference refinements seemed to facilitate choices. To test this, we regressed RT and confidence, separately, on pre-choice overall value difference and overall value certainty difference, as well as SoA and SoAC. We found that all MFX coefficients in both regressions were significantly different than zero in the expected directions (Figure S4; Table S3). Notably, the regression coefficients for SoA and SoAC resembled those for pre-choice overall value difference and overall value certainty difference, supporting the view that SoA measures a true increase in overall value difference and SoAC measures a true increase in overall value certainty difference before choice deliberation is concluded.


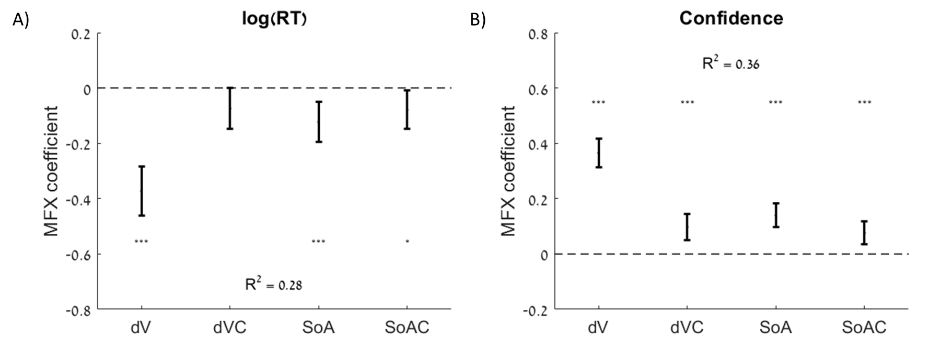


**Figure S4: Explanatory power of value estimates, certainty, and spreading of alternatives on response time and confidence.** Predictive effect of the difference in overall value (dV), difference in overall value certainty (dVC), spreading of alternatives (SoA), and spreading of alternatives’ certainty (SoAC) of the options on each trial on response time (RT; panel A) and confidence (panel B). Error bars represent 95% confidence intervals. Significance stars: * *p* < .05, ** *p* < .01, *** *p* < .001.

**Table S3.** Effects of rating estimates, certainty, and spreading of alternatives on response time and confidence.

|  | log(RT) | Confidence |
| --- | --- | --- |
| *Overall value difference (dV)* | -0.37 (*p* < .001) | 0.37 (*p* < .001) |
| *Overall value certainty difference (dVC)* | -0.07 (*p* = .052) | 0.10 (*p* < .001) |
| *Spreading of alternatives (SoA)* | -0.12 (*p* < .001) | 0.14 (*p* < .001) |
| *Spreading of alternatives’ certainty (SoAC)* | -0.08 (*p* = .027) | 0.08 (*p* < .001) |
|  | R^2^ = 0.28 | R^2^ = 0.36 |

This table lists the mixed-effects regression coefficients from three separate regressions using either choice consistency, response time, or confidence as the dependent variable. All regressions included random intercepts and slopes for each variable by participant.

1. **Data summary**

Below, we provide graphical summaries of all of the empirical variables that we examined in this study. We provide histograms for each variable, separately for data from Experiment 1, Experiment 2 based on pre-choice ratings, and Experiment 2 based on post-choice ratings. We then provide matrices showing the correlations between all rating variables and between all choice variables, separately for data from Experiment 1, Experiment 2 based on pre-choice ratings, and Experiment 2 based on post-choice ratings.


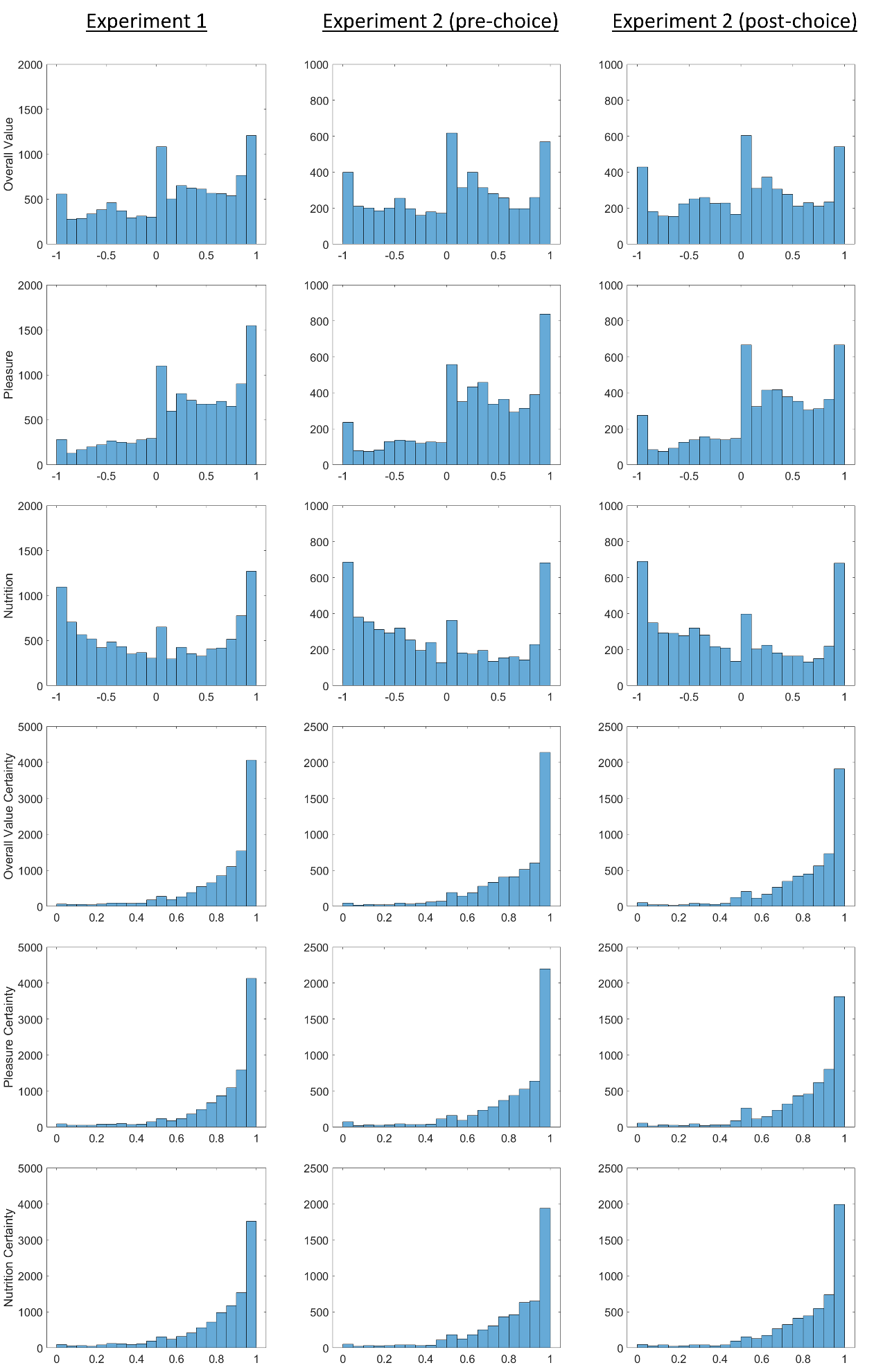


**Figure S5: Empirical distributions of ratings and certainty.** Histograms of the rating (first three rows) and certainty (last three rows) data from Experiment 1 (first column), Experiment 2 pre-choice (second column), and Experiment 2 post-choice (third column).


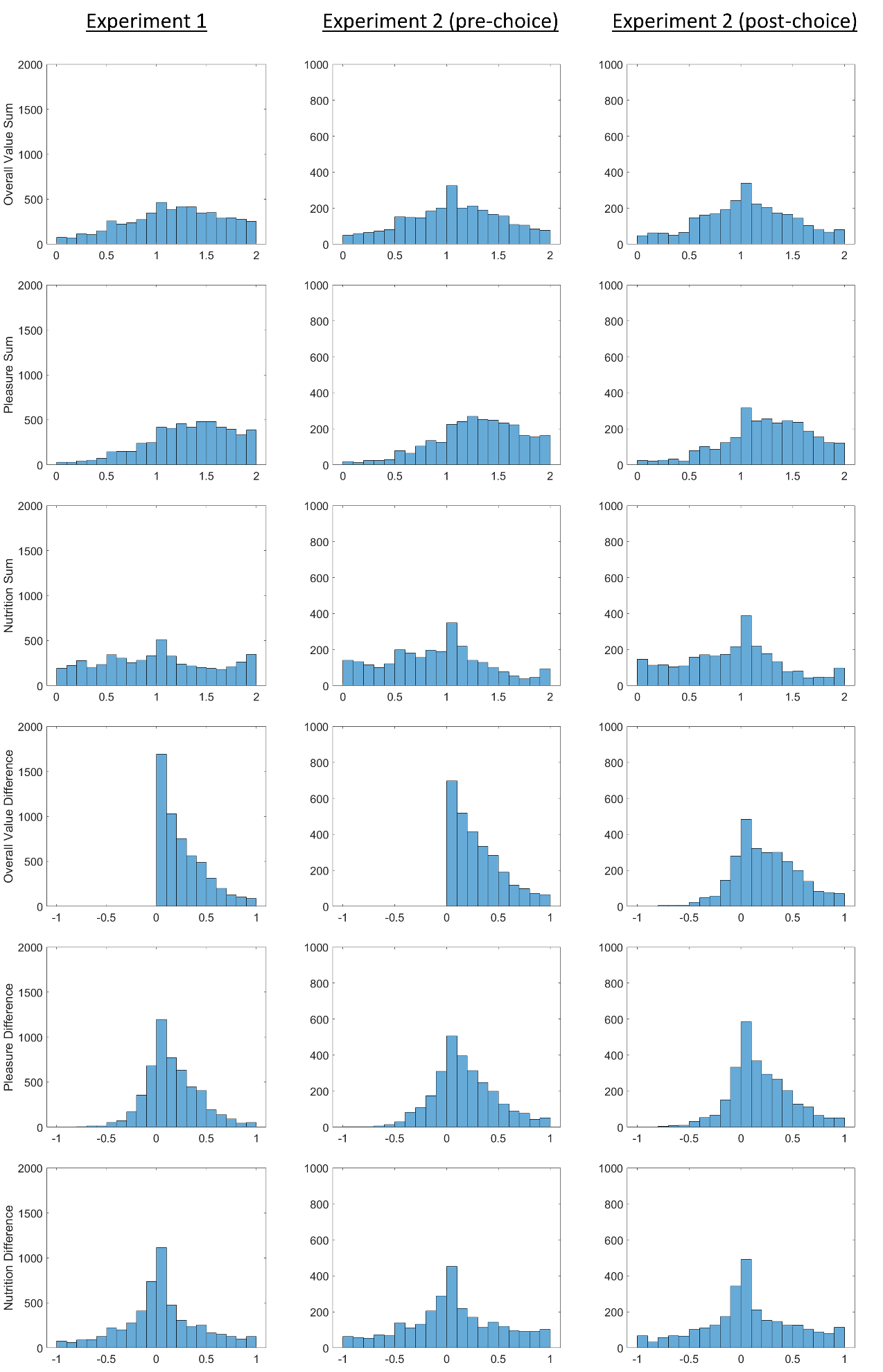


**Figure S6: Empirical distributions of rating sums and differences within choice pairs.** Histograms of the rating sums (first three rows) and differences (last three rows) data from Experiment 1 (first column), Experiment 2 pre-choice (second column), and Experiment 2 post-choice (third column).


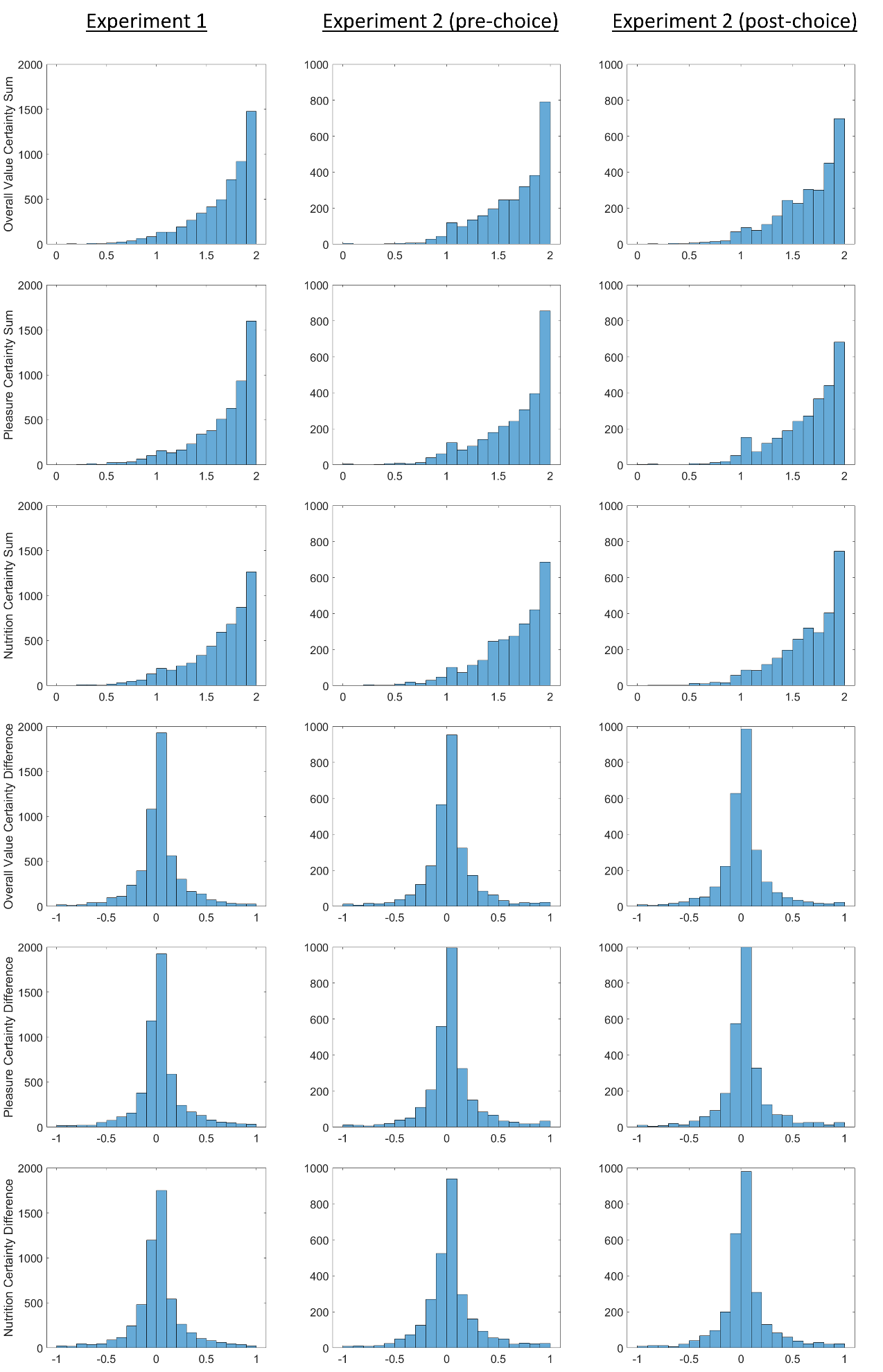


**Figure S7: Empirical distributions of certainty sums and differences within choice pairs.** Histograms of the certainty sums (first three rows) and differences (last three rows) data from Experiment 1 (first column), Experiment 2 pre-choice (second column), and Experiment 2 post-choice (third column).


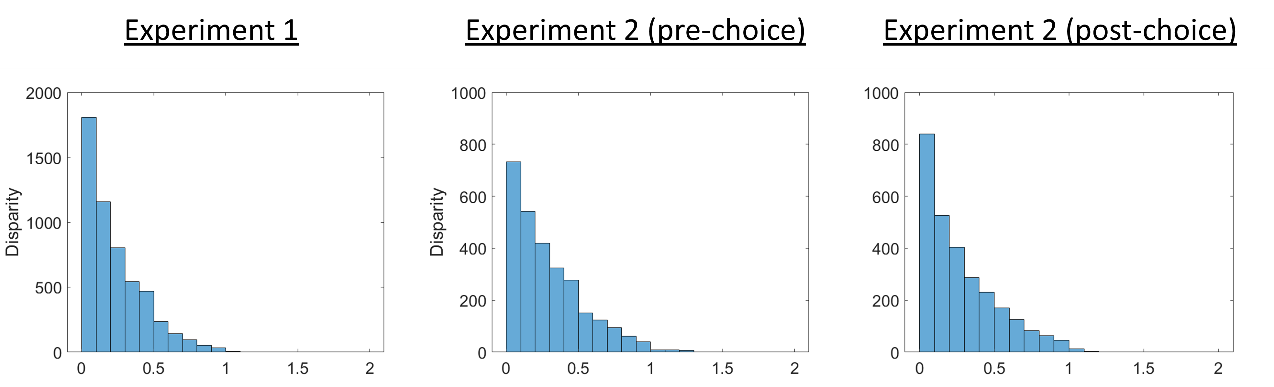


**Figure S8: Empirical distributions of attribute disparity within choice pairs.** Histograms of the attribute disparity data from Experiment 1 (first column), Experiment 2 pre-choice (second column), and Experiment 2 post-choice (third column).


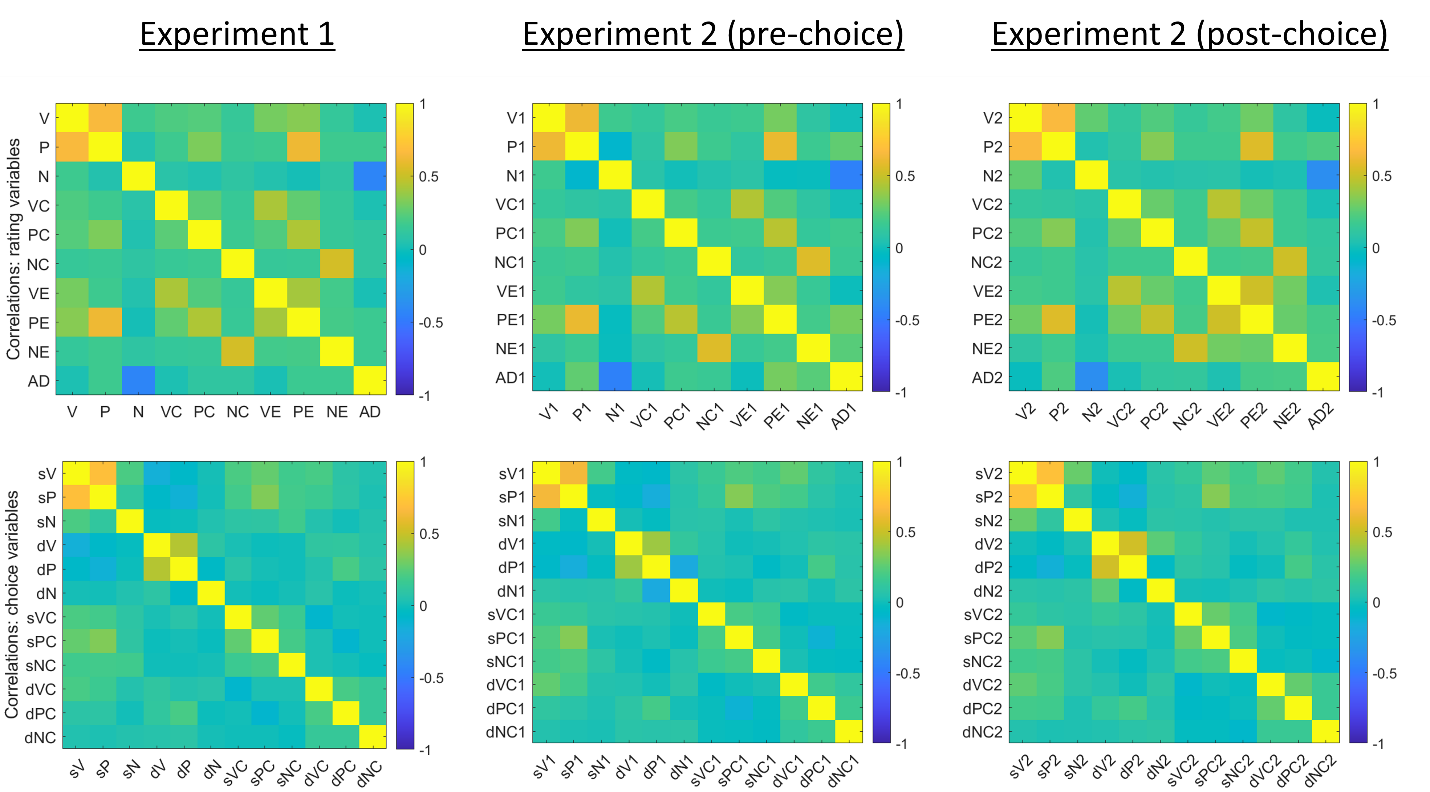


**Figure S9: Correlations between variables.** Matrices of the correlation between each of the variables from Experiment 1 (first column), Experiment 2 pre-choice (second column), and Experiment 2 post-choice (third column). The top row contains variables related to the rating task, the bottom row contains variables related to the choice task.
